# *In silico* screening for human norovirus antivirals reveals a novel non-nucleoside inhibitor of the viral polymerase

**DOI:** 10.1038/s41598-018-22303-y

**Published:** 2018-03-07

**Authors:** Salvatore Ferla, Natalie E. Netzler, Sebastiano Ferla, Sofia Veronese, Daniel Enosi Tuipulotu, Salvatore Guccione, Andrea Brancale, Peter A. White, Marcella Bassetto

**Affiliations:** 1 School of Pharmacy and Pharmaceutical Sciences, Cardiff University, Cardiff, United Kingdom; 2 School of Biotechnology and Biomolecular Sciences, University of New South Wales, Sydney, NSW Australia; 3 Dipartimento di Scienze del Farmaco, Università degli Studi di Catania, Catania, Italy

## Abstract

Human norovirus causes approximately 219,000 deaths annually, yet there are currently no antivirals available. A virtual screening of commercially available drug-like compounds (~300,000) was performed on the suramin and PPNDS binding-sites of the norovirus RNA-dependent RNA polymerase (RdRp). Selected compounds (n = 62) were examined for inhibition of norovirus RdRp activity using an *in vitro* transcription assay. Eight candidates demonstrated RdRp inhibition (>25% inhibition at 10 µM), which was confirmed using a gel-shift RdRp assay for two of them. The two molecules were identified as initial hits and selected for structure-activity relationship studies, which resulted in the synthesis of novel compounds that were examined for inhibitory activity. Five compounds inhibited human norovirus RdRp activity (>50% at 10 µM), with the best candidate, **54**, demonstrating an IC_50_ of 5.6 µM against the RdRp and a CC_50_ of 62.8 µM. Combinational treatment of **54** and the known RdRp site-B inhibitor PPNDS revealed antagonism, indicating that **54** binds in the same binding pocket. Two RdRps with mutations (Q414A and R419A) previously shown to be critical for the binding of site-B compounds had no effect on inhibition, suggesting **54** interacts with distinct site-B residues. This study revealed the novel scaffold **54** for further development as a norovirus antiviral.

## Introduction

Human norovirus is responsible for over 200,000 global deaths, USD $60 billion in societal costs, and USD $4.2 billion in health expenditure each year^1^. While norovirus-induced illness is usually self-limiting in healthy individuals, the disease can be severe or life threatening in immunocompromised and immunosuppressed patients^2,3^, neonates^4^, young children^5,6^ and elderly populations^7,8^. As such, a significant research effort has been invested to identify antiviral candidates for the treatment of norovirus infections and for prophylactic use in outbreak settings.

Several factors have hampered norovirus antiviral research, including the inability to successfully propagate human norovirus in cell culture until recently^9,10^. However, the recently developed human norovirus culture systems are not readily amenable to antiviral screening due to low replication levels, with only 100- to 1000-fold increases in viral titres^9,10^, and the systems’ complexities and costs. Most norovirus antiviral research currently relies on the closely related murine norovirus (MNV), for screening potential human antivirals due to its robust and reliable culture, and available mouse models^11^.

**Figure 1:**
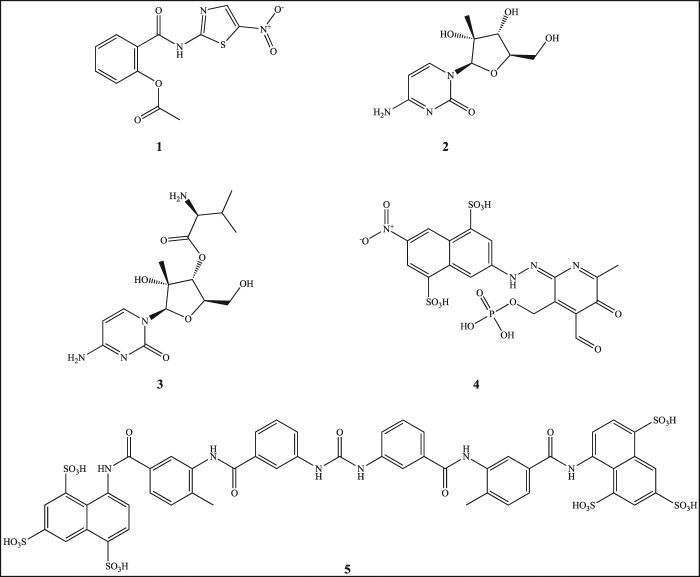
Norovirus inhibitors in pre-clinical studies. Nitazoxanide (**1**), 2′-C-methylcytidine (**2**), valopicitabine (**3**), PPNDS (**4**), suramin (**5**).

The only norovirus antiviral in clinical trials to-date is the broad-spectrum antimicrobial compound nitazoxanide^12^ (Fig. 1). This compound has successfully treated one immunosuppressed patient^13^, and reportedly reduced the duration of symptoms in both norovirus and rotavirus infected patients^12^. However, the exact mode of action of nitazoxanide against norovirus is still unknown, and more research is required to establish its antiviral target.

There are a small number of human norovirus antiviral candidates in preclinical development. The majority of these therapeutic candidates target the viral RNA-dependent RNA polymerase (RdRp)^14–17^, or the viral protease^18–20^, due to their critical role in viral replication, and their lack of cellular homologues^21,22^. RdRp-targeted antivirals fall into two classes; nucleoside analogues (NAs) and non-nucleoside inhibitors (NNIs)^23^. NA prodrugs can be phosphorylated after cellular uptake, then incorporated into the growing viral nucleic acid chain by the RdRp^24^. These antivirals work by competing with incoming nucleoside triphosphates (NTPs) to cause either chain termination^25^, or lethal mutagenesis of the progeny virus^26^. NAs have proven successful in the treatment of several viral infections including hepatitis B virus^27^, hepatitis C virus (HCV)^28^, herpesviruses^29^ and human immunodeficiency virus (HIV)^30^. However, due to their mode of action, they frequently result in off-target side effects^31,32^, and no NAs have yet reached clinical trials for norovirus treatment. One NA, 2′-C-methylcytidine (2CMC) (Fig. 1), inhibited the human norovirus RdRp, with a reported half maximal inhibitory concentration (IC_50_) of 2.5 µM^15^, and a half maximal effective concentration (EC_50_) of 8.2 µM in cell culture against the human norovirus replicon^15^. More recently, 2CMC was also found to be effective against human norovirus cultured in the B-cell culture system, with a reported EC_50_ of 0.3 µM^33^. However, despite these promising preclinical results, development of the oral 2CMC prodrug (valopicitabine) (Fig. 1) for the treatment of HCV patients was terminated after gastrointestinal effects were reported, and further development against other viruses including human norovirus is now unlikely.

Unlike NAs, NNIs generally bind to allosteric pockets on the viral polymerase to prevent conformational changes necessary for transcription^34^. NNIs have been clinically approved for treating HIV^35^ and HCV^36^ infections, however, while several potential norovirus NNIs have been identified in pre-clinical studies^14,16,17,37–39^, none are in clinical trials for the treatment of human norovirus infections. Amongst these pre-clinical candidates is the anthelmintic drug suramin (Fig. 1), which demonstrated potent *in vitro* inhibitory activity against the human norovirus RdRp in enzymatic assays with an IC_50_ of 27.0 nM^37^. Similarly, the suramin derivative, pyridoxal-5′-phosphate-6-(2′-naphthylazo-6′-nitro-4′,8′-disulfonate) tetrasodium salt (PPNDS) (Fig. 1), has also been shown to inhibit human norovirus RdRp activity *in vitro*, with an IC_50_ of 0.45 µM^17^. Recently, PPNDS was reported to exhibit broad-spectrum antiviral activity against other calicivirus RdRps^16^. However, neither of these compounds are likely to be suitable for the treatment of human norovirus infections, as both suramin and PPNDS display poor cell membrane permeability^17,40^, suramin induces severe side-effects^41,42^, and PPNDS is known to have non-specific, off-target activity^43^.

The human norovirus polymerase forms the canonical ‘right-hand’ RdRp structure, with fingers, palm and thumb domains^44^. To-date, three NNI binding sites have been identified on the norovirus RdRp, including the NTP pathway, site-A in the fingers domain and site-B in the thumb domain^17,37^. Co-crystallisation studies of the norovirus RdRp and suramin identified its binding pocket in the NTP access pathway, between the fingers and thumb domains^37^. Site-A lies within a positively charged cleft for NTP traversal, but due to highly flexible amino acid side-chains it is less suitable as an NNI target, due to challenges for structure-based antiviral design^17^. However, site-B is a highly conserved binding site within the thumb region and can be exploited for both structure-activity and targeted *de-novo* antiviral design of novel compounds against human norovirus^16^.

This study aimed to identify promising chemical scaffolds for antiviral development targeting the human norovirus RdRp. To achieve this aim, a series of structure-based and ligand-based virtual screenings were carried out on approximately 300,000 commercially available compounds with favourable drug-like properties, to identify binding capabilities focused primarily on the suramin and PPNDS binding-sites of the norovirus RdRp. This *in silico* screening identified 62 compounds that were examined for inhibition of norovirus RdRp activity using polymerase assays. Two molecules were found to have an interesting RdRp inhibitory activity and were selected for a structure-activity relationship study synthesising new chemical derivatives. Among the newly prepared compounds, we identified a lead candidate site-B binder, which inhibited the human norovirus RdRp at a low micromolar concentration. This study revealed a novel NNI that presents a promising scaffold for further chemical enhancement, to improve potency and specificity, in the pursuit of effective norovirus antivirals.

## Results and Discussion

### Molecular Modelling: hit identification

Two different virtual screening approaches have been used for the initial selection of potential hit compounds: a structure-based virtual screening and a ligand-based virtual screening. While the first approach takes into consideration the protein structure and the ligand spatial disposition in relation to the active site (pharmacophore features), the ligand-based methodology compares the shapes of known ligands with unknown ones, evaluating two-dimensional similarities (i.e. same functional groups) or three-dimensional similarities (molecule occupational volume) without considering the protein structure^45^. The molecules selected with the two methods were then subjected to different molecular docking evaluations described below.

### Structure-based virtual screening

The norovirus RdRp was used to perform a structure-based virtual screening of the SPECS library, a database of ~300,000 commercially available compounds^46^. In particular, two screens were conducted using two different RdRp crystal structures: the MNV RdRp co-crystallised with suramin (PDB ID: 3UR0, 62% identity with the human norovirus RdRp)^37^ and the human RdRp co-crystallised with PPNDS (PDB ID: 4LQ3)^17^. Suramin inhibits human norovirus and MNV RdRp activity through a mixed inhibition mode (nucleic acid and GTP) and occupies site-A. Site-A encompasses the access route for the incoming NTP that is linked to the nascent RNA chain. PPNDS inhibits human norovirus RdRp activity by blocking the access of both the ssRNA template and the NTPs, and occupies the inner pocket of site-B, which is located in the RdRp thumb domain^17^. The two binding sites are very close to each other and overlap in the central part of the protein structure.

**Figure 2:**
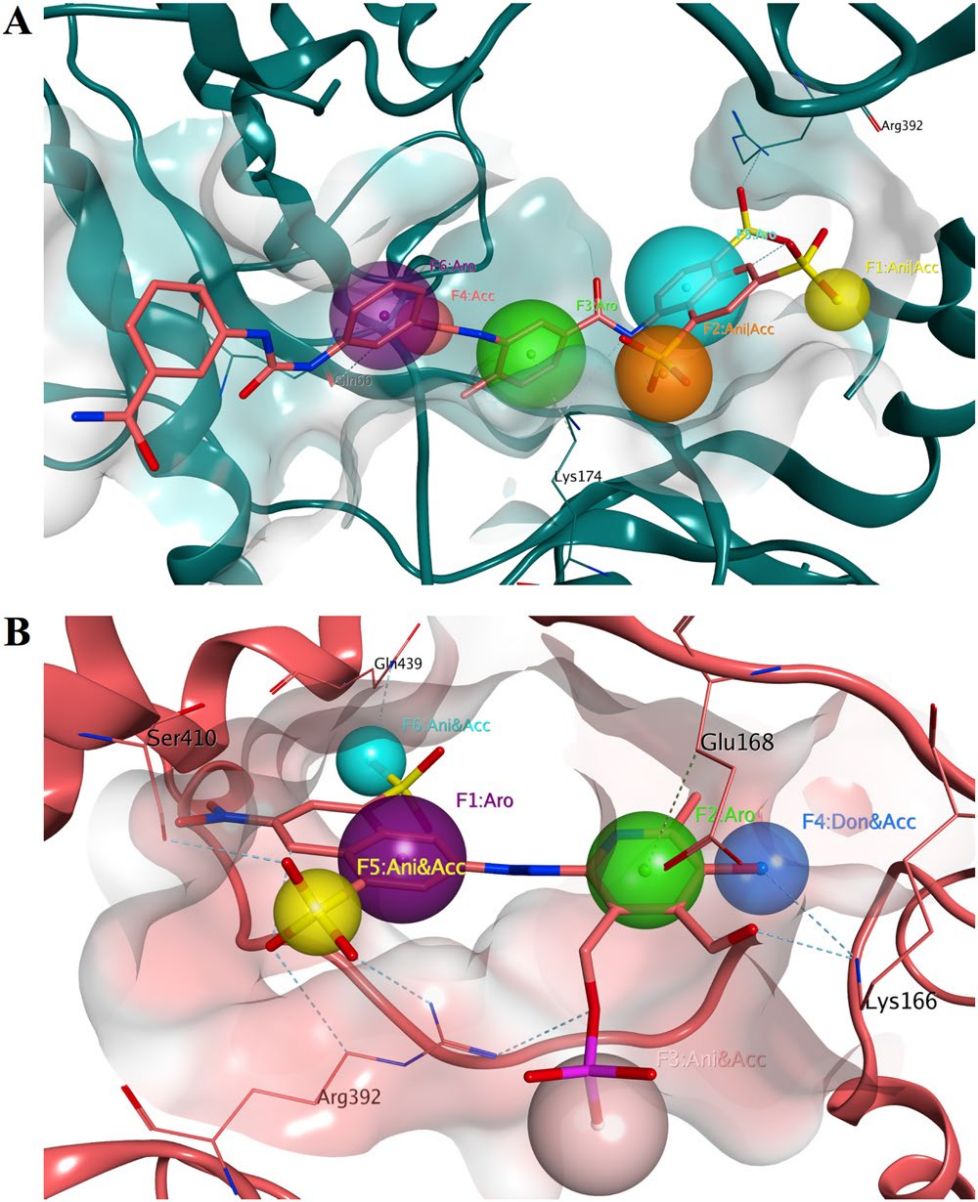
Pharmacophore models used for the structure-based virtual screening. (**A**) Model based on suramin binding to the MNV RdRp 3UR0 crystal structure. The model consists of three aromatic features (purple, green and turquoise), one hydrogen bond acceptor (pink) and two hydrogen bond acceptors or anionic groups (orange and yellow). Only the interacting portion of suramin has been considered for the pharmacophore creation. (**B**) Model based on PPNDS binding to the human norovirus RdRp 4LQ3 crystal structure. The model consists of two aromatic features (purple and green), three hydrogen bond acceptors and anionic groups (pink, turquoise and yellow) and one hydrogen bond donor and acceptor. Exclusion volumes are hidden for clarity. The two binding site areas are represented as molecular surface. The MNV RdRp structure is shown as dark green ribbon, while the human norovirus RdRp is represented as salmon ribbon.

The SPECS database of compounds was pre-filtered using a pharmacophore model based on the suramin binding site in one case and on the PPNDS binding site in the second case (Fig. 2A,B). The required structural features of the suramin pharmacophore were identified in 4838 structures, whereas 5206 matched the PPNDS model. These two sub-libraries were then docked using PLANTS software^47^ on the suramin and on the PPNDS binding sites, respectively. The docking results (docking poses) obtained for each binding site were subsequently re-scored using other two different docking programs: LeadIT-FlexX and Glide XP^48,49^. Applying an internal *consensus* score procedure, a methodology validated by our research group in the past^50^, the values of the three different scoring functions for each docking pose were analysed together. Only the docking poses falling in the top 25% of the score value range in all the three scoring functions were considered as hits. 799 compounds were selected for the suramin pocket, 755 for the PPNDS. A final selection was made by visually inspecting the docking poses of the different molecules and their potential binding to the target sites (see experimental section). 19 derivatives were selected for the suramin binding site and 20 for the PPNDS site. The molecules were purchased and screened for inhibition of norovirus RdRp activity using a transcription enzyme assay.

### Ligand-based virtual screening

**Figure 3:**
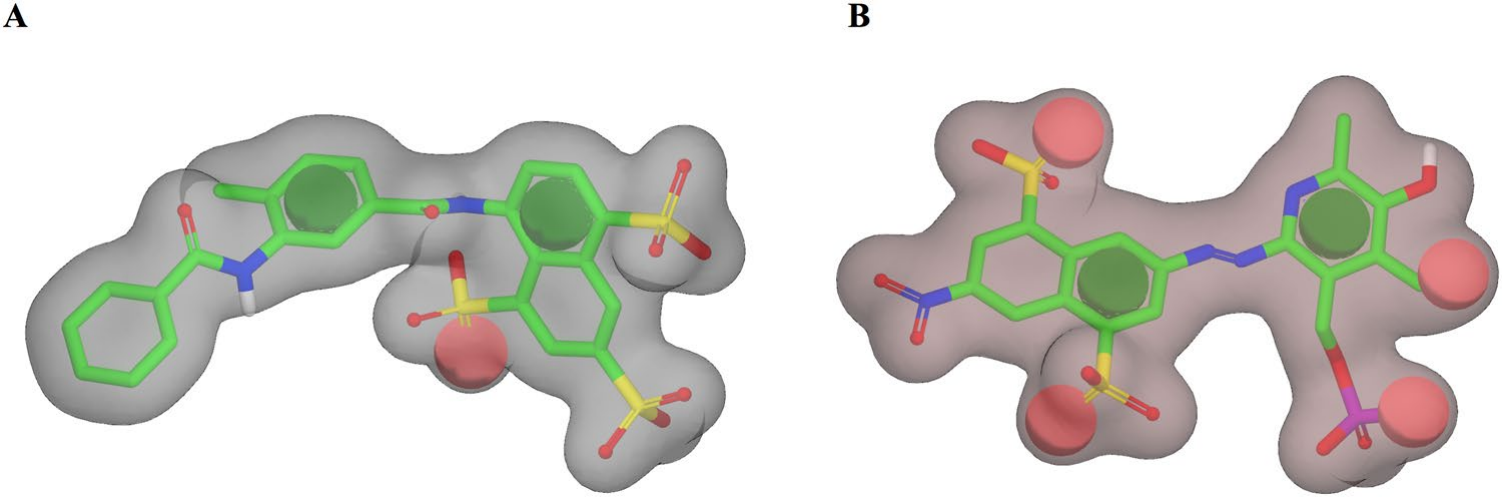
Queries generated using vROCS. (**A**) Query generated using the partial structure of suramin. (**B**) Query generated using the structure of PPNDS. The overall molecular shape of both the inhibitors is represented as a grey surface, while the chemical features considered are an H-bond acceptor (red sphere) and hydrophobic/aromatic centres (green spheres).

A shape-based screening of the SPECS library, previously prepared with the Omega conformations generator program, was performed using ROCS 3D^51^. Two different queries were generated: one based on the partial structure of suramin, and a second prepared on the PPNDS structure (Fig. 3A,B). In both cases, the occupational volume of the two molecules was generated and the relevant chemical properties of each ligand were included as pharmacophore features. The two queries were used to pre-filter the SPECS database; 6563 molecules matched the shape and electrostatic features (Tanimoto combo score) of suramin, whereas 6571 resulted from the PPNDS query. Also in this screen, the two new sub-libraries were then docked using PLANTS software on the suramin and on the PPNDS binding sites respectively. After the *consensus* score procedure and a final visual inspection (see experimental section), three compounds were selected for suramin and 20 for PPNDS. The new derivatives were purchased and biologically evaluated.

## Biological Evaluation

### Two candidates inhibit norovirus RdRp activity

**Figure 4:**
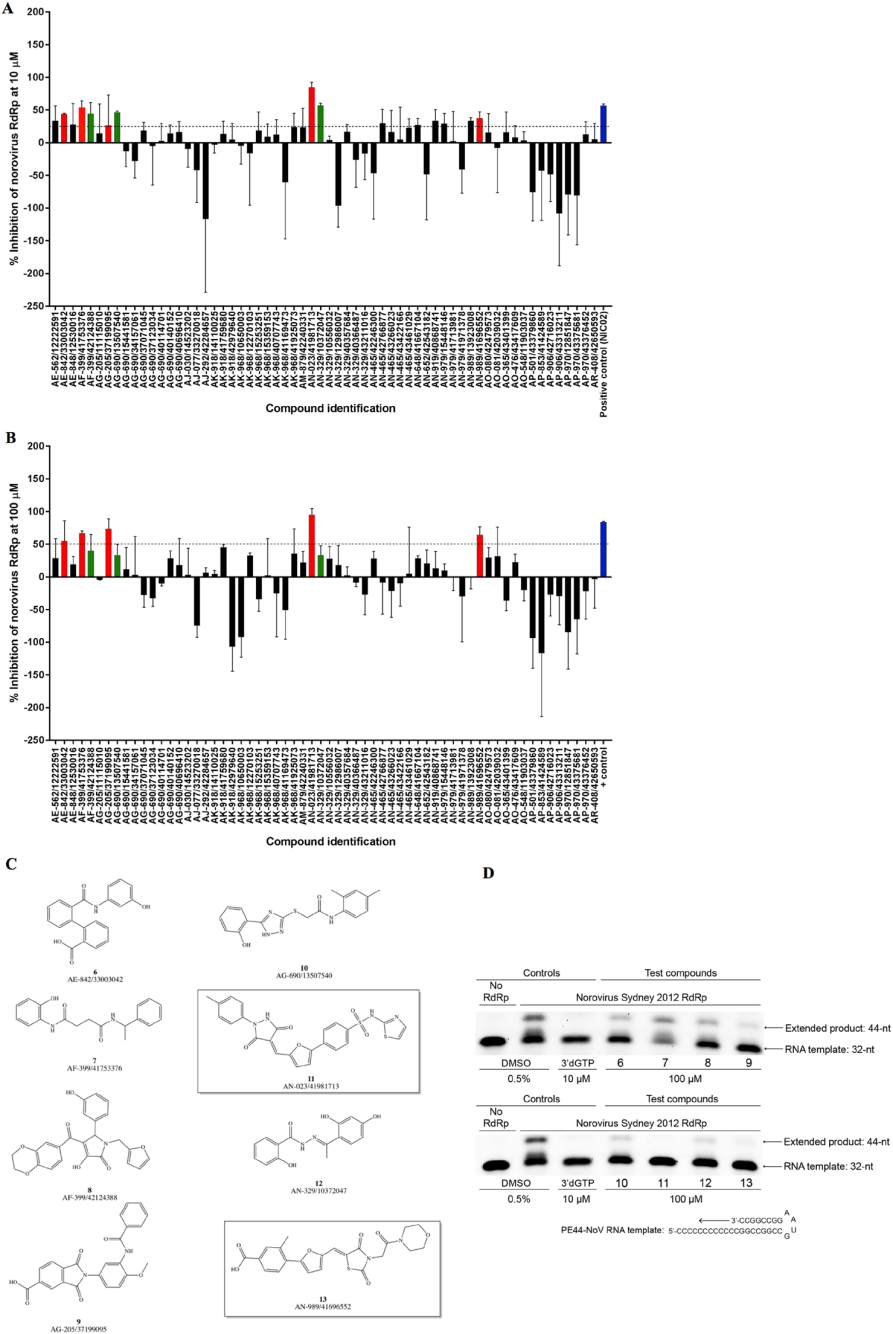
Initial screen for inhibition of human norovirus Sydney 2012 RdRp activity. The effects of 62 compounds were examined against norovirus RdRp activity using a fluorescent activity assay at 10 µM (**A**) and 100 µM (**B**). Five compounds that exhibited more than 25% inhibition at 10 µM and 50% inhibition at 100 µM (red bars) and three soluble compounds that exhibited more than 25% inhibition at both concentrations (green bars) were chosen for further assessment with a counter-screen gel-shift RdRp assay. The positive control (blue bars) is a previously reported norovirus NNI RdRp inhibitor NIC02 (10 µM in (**A**) and 100 µM in (**B**)^14^. The mean values of triplicate datasets with standard deviation are shown. (**C**) Structure of the eight most potent compounds with **11** and **13** highlighted. (**D**) A counter-screen gel-shift assay was used to confirm norovirus RdRp inhibitory activity of hit compounds. The eight compounds were examined for inhibition of primed elongation activity. PE44-NoV RNA templates (32 nucleotides) were extended (44 nucleotides) by the RdRp in the absence of any test compounds (0.5% DMSO [vol/vol] negative control) or with test compounds at a fixed concentration of 100 µM. The nucleoside analogue 3′-deoxyguanosine-5′-triphosphate is used as a positive control (10 µM) to demonstrate complete inhibition, and no RdRp was used as a negative control. Full-length gels are presented in Supplementary Fig. S2.

The 62 compounds identified *in silico* were screened for inhibition of norovirus RdRp activity using an *in vitro* fluorescent, *de novo* RdRp assay^52^. Compounds were tested at fixed concentrations of 10 µM and 100 µM, and compared to mock treated samples containing the vehicle only (0.5% DMSO [vol/vol]) (Fig. 4A,B). Of the 62 compounds examined, five (Fig. 4C) exhibited more than 25% inhibition of human norovirus RdRp activity at 10 µM (Fig. 4A) and more than 50% at 100 µM (Fig. 4B). For certain compounds, significant insolubility was observed, coupled with either inherent fluorescence quenching (positive y-axis values on Fig. 4A,B) or enhancement of the fluorescence signal (negative y-axis values). Therefore, a secondary assay that does not rely on relative fluorescence was employed as an effective counter-screen to exclude false positives and compounds that showed enhanced fluorescence.

In addition, some compounds (i.e. AJ-077/33270018 and AP-970/12851847) exhibited insolubility, innate fluorescence and even apparent RdRp enhancement at 100 µM (negative y-axis values Fig. 4B). Therefore, they were counter-screened at 100 µM in a secondary gel-based assay (Supplementary Fig. S1). The putative RdRp activity enhancement observed in the primary fluorescent assay was not seen in the gel-based assay, confirming compound insolubility and innate fluorescence that was likely affecting assay results.

To confirm RdRp inhibition and to exclude any potential false positives from the first fluorescence RdRp assay, the eight selected compounds (Fig. 4C) were then assessed using a gel-shift enzyme activity assay. These eight compounds included five that demonstrated dose-dependent inhibition (red bars, compounds **6**, **7**, **9**, **11** and **13**) reaching over 25% inhibition at 10 µM and more than 50% inhibition at 100 µM. Three additional compounds (compounds **8**, **10** and **12**) that reached more than 25% inhibition at both 10 and 100 µM were selected for counter-screening. The three compounds (green bars) were examined using the gel-based assay due to the unusual behaviour of demonstrating higher inhibition at 10 µM compared to 100 µM, potentially due to solubility issues. Two additional compounds (AE-562/12222591 and AK-968/41925073) demonstrated more than 25% inhibition at 10 and 100 µM but were not pursued further as they both precipitated heavily in 100% DMSO and therefore lacked the desirable drug-like property of solubility.

In this assay, a 32 nucleotide RNA template (PE44-NoV) is extended to 44 nucleotides in the presence of an active RdRp^16^. The NA 3′-deoxyguanosine-5′-triphosphate (3′dGTP) was used as a positive control and exhibited complete inhibition of transcription at 10 µM. Inhibition of primed elongation of the PE44-NoV template was examined at a fixed concentration of 100 µM (Fig. 4D). In the absence of inhibition (0.5% DMSO vehicle only) the RNA template was extended to 44 nucleotides (Fig. 4D), evidenced by an upward gel shift and the appearance of the second, higher band. Only two of the eight compounds, **11** and **13**, exhibited complete inhibition of norovirus RdRp activity at 100 µM (Fig. 4D), while the remaining six compounds (**6–10 and 12**) only demonstrated partial inhibition of primed elongation activity, and were excluded from further development. Based on these results, we decided to focus our attention on these two molecules.

## Chemistry

Due to their confirmed inhibitory activity against the RdRp polymerases, the structures of **11** and **13** were chosen as starting point for the preparation of a series of analogues, with which to explore the structure-activity relationships associated with the two scaffolds.

### Hit 13 modified analogues

**13** is characterized by a methylbenzoic acid portion and a morpholine ring part linked by a central furan-2-yl-methylene-thiazolidine-2,4-dione core. We focused our initial synthetic efforts on the two lateral portions of the molecule, without modifying the central core, in order to investigate the role of these two parts in the RdRp inhibitory activity.

**Figure 5:**
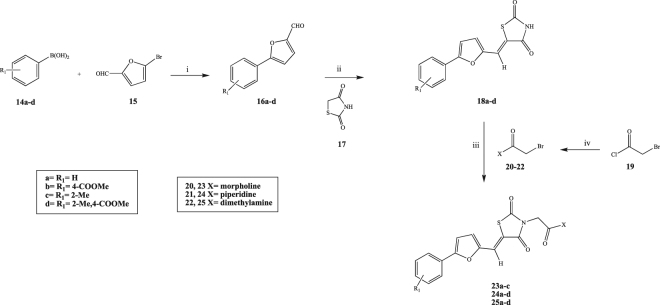
General synthetic procedure followed for the preparation of 23a-c, 24a-d, 25a-d. (i) Na_2_CO_3_, Pd(PPh_3_)_2_, THF/H_2_O, r.t. 8 h, 60 °C 12 h; (ii) β-alanine, AcOH, 100 °C 1 h; (iii) K_2_CO_3_, DMF, r.t. o.n., 60 °C 72 h; (iv) secondary amine, DIPEA, DCM, 0 °C to r.t. 20 min.

The synthetic pathway reported in Fig. 5 was obtained after several optimizations of reaction methods and routes. The differently substituted phenylfuran-2-carbaldehydes **16a–d** were prepared by reacting the corresponding phenylboronic acids (**14a–d**) with 5-bromofuran-2-carbaldehyde (**15**) in a mix of tetrahydrofuran/water, through a Suzuki coupling reaction using sodium carbonate (Na_2_CO_3_) as base and tetrakis (Pd[Ph]_3_]_4_) as catalyst modifying a reported procedure^53^. The desired products were obtained in a very good yield range (62–98%) after flash column chromatography purification. A Knoevenagel condensation was exploited for the preparation of derivatives **18a–d** by reacting compounds **16a–d** with thiazolidine-2,4-dione (**17**), using β-alanine as catalyst and acetic acid as solvent^53^. In the final step, compounds **18a–d** were converted into the corresponding final products **23a–c, 24a–d, 25a–d** by reacting them with **20–22** in dimethylformamide (DMF) at 60 °C for 72 h using potassium carbonate (K_2_CO_3_) as base^54^. The final compounds were obtained in a good yield range (42–76%) after either re-crystallization or flash column chromatography purification. Compounds **20–22** were previously prepared by reacting 2-bromoacetyl chloride (**19**) with different secondary amines in dichloromethane (DCM) and *N,N*-diisopropylethylamine (DIPEA) as base^55^. Synthetic efforts were made to achieve good yields for this step by monitoring the reaction temperature and evaluating different bases. Following this synthetic route, 11 derivatives of **13** were prepared.

As reported above, **13** presents a free acid group, therefore a basic hydrolysis of **23b** with LiOH in water and methanol at room temperature was attempted. Unfortunately, NMR analysis revealed complete degradation of the starting material with the opening of the thiazolidinedione ring, revealing instability for these compounds in basic environments. A second attempt was made by performing an acid hydrolysis in HCl 6 M increasing the reaction temperature to 100 °C over 3 days. In this case, the desired hydrolysis did not take place and the starting ester was recovered.

**Figure 6:**
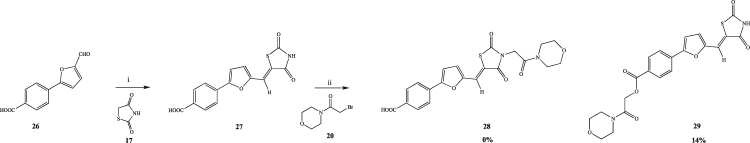
Alternative method for the preparation of the free acid derivative. (i) β-alanine, AcOH, 100 °C 1 h; (ii) K_2_CO_3_, DMF, r.t. o.n., 60 °C 72 h.

In the attempt to find a strategy to prepare the free acid derivatives, the previously used synthetic pathway was slightly modified, as reported in Fig. 6, by performing the ester hydrolysis of **16b** as second step after the Suzuki coupling, obtaining **26**. Hydrolysed **26** was then condensed with **17** to give **27**. In the last step, the reaction between **27** and **20** did not yield the desired product **28**, the unreacted staring materials were recovered, along with small amounts of a minor by-product, **29**, in which the substitution of the bromide compound occurred on the carboxylic acid portion of the molecule instead of the thiazolidinedione ring, as also reported by Zhang *et al*.^56^. The free acid seems to interfere with the S_N_2 reaction and a different approach was therefore investigated.

**Figure 7:**
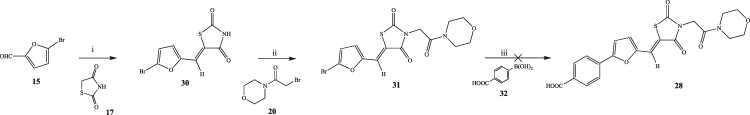
Alternative method for the preparation of the free acid derivatives (i) β-alanine, AcOH, 100 °C 1 h; (ii) K_2_CO_3_, DMF, r.t. o.n., 60 °C 72 h; (iii) Na_2_CO_3_, Pd(PPh_3_)_2_, THF/H_2_O, r.t. 8 h, 60 °C 12 h.

Figure 7 shows the planned modifications to the original synthetic pathway to prepare **28**. In this case, the Knoevenagel condensation was performed as a first step giving **30**. Nucleophilic substitution with **20** gave the central furan-2-yl-methylene-thiazolidine-2,4-dione core with the attached morpholine ring portion (**31**). For the last step, the Suzuki coupling between the 4-boronobenzoic acid **32**, previously prepared starting from its ester derivative (**14b**), and **31** was attempted, but no desired product was obtained, recovering the unreacted boronic acid and observing degradation of the thiazolidinedione ring.

**Figure 8:**
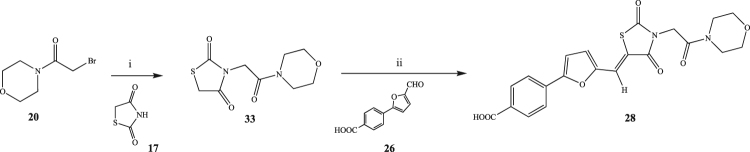
Preparation of the free acid derivative 28 (i) K_2_CO_3_, DMF, r.t. o.n., 60 °C 72 h; (ii) β-alanine, AcOH, 100 °C 1 h.

**Figure 9:**
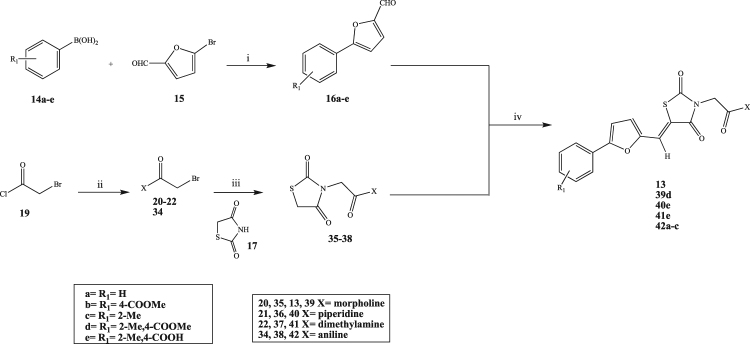
General synthetic procedure followed for the preparation of 13, 39d, 40e, 41e, 42a-c. (i) Na_2_CO_3_, Pd(PPh_3_)_2_, THF/H_2_O, r.t. 8 h, 60 °C 12 h; (ii) secondary amine, DIPEA, DCM, 0 °C to r.t. 20 min; (iii) K_2_CO_3_, DMF, r.t. o.n., 60 °C 72 h or NaH, THF, 60 °C, 24 h; (iv) β-alanine, AcOH, 100 °C 1 h.

A final and alternative method for the preparation of the desired free acid **28** was developed, considering the target molecule as an assembly of two portions: the 4-(5-formylfuran-2-yl)benzoic acid (**26**), prepared by Suzuki coupling (Fig. 6), and the 3-(2-morpholino-2-oxoethyl)thiazolidine-2,4-dione (**33**), obtained by the S_N_2 reaction between **17** and **20**. The two portions were then linked together in the last step through a Knoevenagel condensation affording the desired product **28** with the free acid (Fig. 8). This new approach was used for the preparation of other seven final compounds, including the original hit **13**, that could be used as reference for the new biological evaluation (Fig. 9). Also for this synthetic pathway, the different steps were optimised to afford a high yield. In particular, for the preparation of the thiazolidine-2,4-dione derivatives (**35–38**), the reaction conditions needed to be changed according to the substituted amine present. In the case of piperidine and dimethylamine derivatives (**36, 37**), the use of sodium hydride (NaH) as base and THF as solvent gave a less impure reaction and a higher yield if compared to the method used for the other derivatives.

### Hit 11 modified analogues

**11** has a central furan-2-ylmethylene-pyrazolidine-3,5-dione core substituted at position 1 of the pyrazolidine with a methyl benzene and at position 5 of the furane ring with a thiazol-2-yl-benzenesulfonamide. A five-step synthetic pathway was developed and optimised and a small family of two new derivatives was prepared in order to initially understand the role of the methyl group and the thiazole ring.

Hydrazides **46–47** were prepared by reacting the corresponding phenylhydrazines (**43–44**) with ethyl-3-chloro-3-oxopropanoate (**45**) in THF and triethylamine (Et_3_N), following a reported methodology^57^. **46–47** were then converted into the corresponding 1-phenylpyrazolidine-3,5-dione **48–49** through an ester displacement reaction in the presence of sodium hydroxide (NaOH) and ethanol (EtOH)^57^. 4-Bromobenzene-1-sulfonyl chloride (**50**) and aniline **51** in pyridine gave sulfonamide **52**, which was then converted in **53** by a Suzuki coupling. In particular, synthetic efforts were made to find the best conditions for this coupling, since the procedure previously described did not work and only the starting materials were recovered after each attempt, potentially due to an interference of the sulfonamide bond with the catalyst used. After changing solvent systems, reaction temperature, base and phosphine ligands, the best reaction conditions were found using potassium phosphate (K_3_PO_4_) as base, Pd(dppf) as catalyst, water/DMF as solvent and heating in a microwave reactor at 130 °C, which gave the desired product in a good yield (61%).

**Figure 10:**
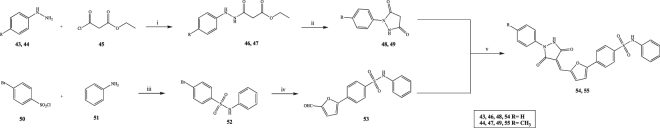
General synthetic procedure followed for the preparation of 54, 55. (i) NEt_3_, THF, r.t. 3 h; (ii) NaOH, EtOH, r.t. 30 min; (iii) pyridine, r.t. o.n.; (iv) K_3_PO_4_, Pd(dppf), H_2_O/DMF, 130 °C 75 min (microwave); (v) AcOH, 120 °C 3 h.

In the last step (Fig. 10), the two portions were linked together by reacting **48–49** and **53** according to a Knoevenagel condensation, giving the desired products **54** and **55**.

## Biological Evaluation

### Three newly prepared compounds inhibit human norovirus RdRp activity

The newly synthesised compounds, including the re-prepared hit **13** and some synthesis intermediates (Table 1), were initially evaluated on norovirus Sydney 2012 RdRp activity *in vitro* using a quantitative fluorescent assay. Compounds were tested at a fixed concentration of 10 μM and compared to the relative activity of mock treated samples.

**Table 1:** Newly prepared compounds evaluated on recombinant norovirus Sydney 2012 RdRp activity *in vitro* assay. S.I. = synthesis intermediate; S.P. = side product.


**Compound**	**R**_**1**_	**X**	**Compound**	**R**_**1**_	**X**
**13**	2-Me, 4-COOH		**25c**	2-Me	
**23a**	H		**25d**	2-Me, 4-COOMe	
**23b**	4-COOMe		**41e**	2-Me, 4-COOH	
**23c**	2-Me		**42a**	H	
**28**	4-COOH		**42b**	4-COOMe	
**39d**	2-Me, 4-COOMe		**42c**	2-Me	
**24a**	H		**18a S.I**.		
**24b**	4-COOMe		**18c S.I**.		
**24c**	2-Me		**27 S.I**.		
**24d**	2-Me, 4-COOMe		**31 S.I**.		
**40e**	2-Me, 4-COOH		**29 S.P**.		
**25a**	H		**54**		
**25b**	4-COOMe		**55**		

**Figure 11:**
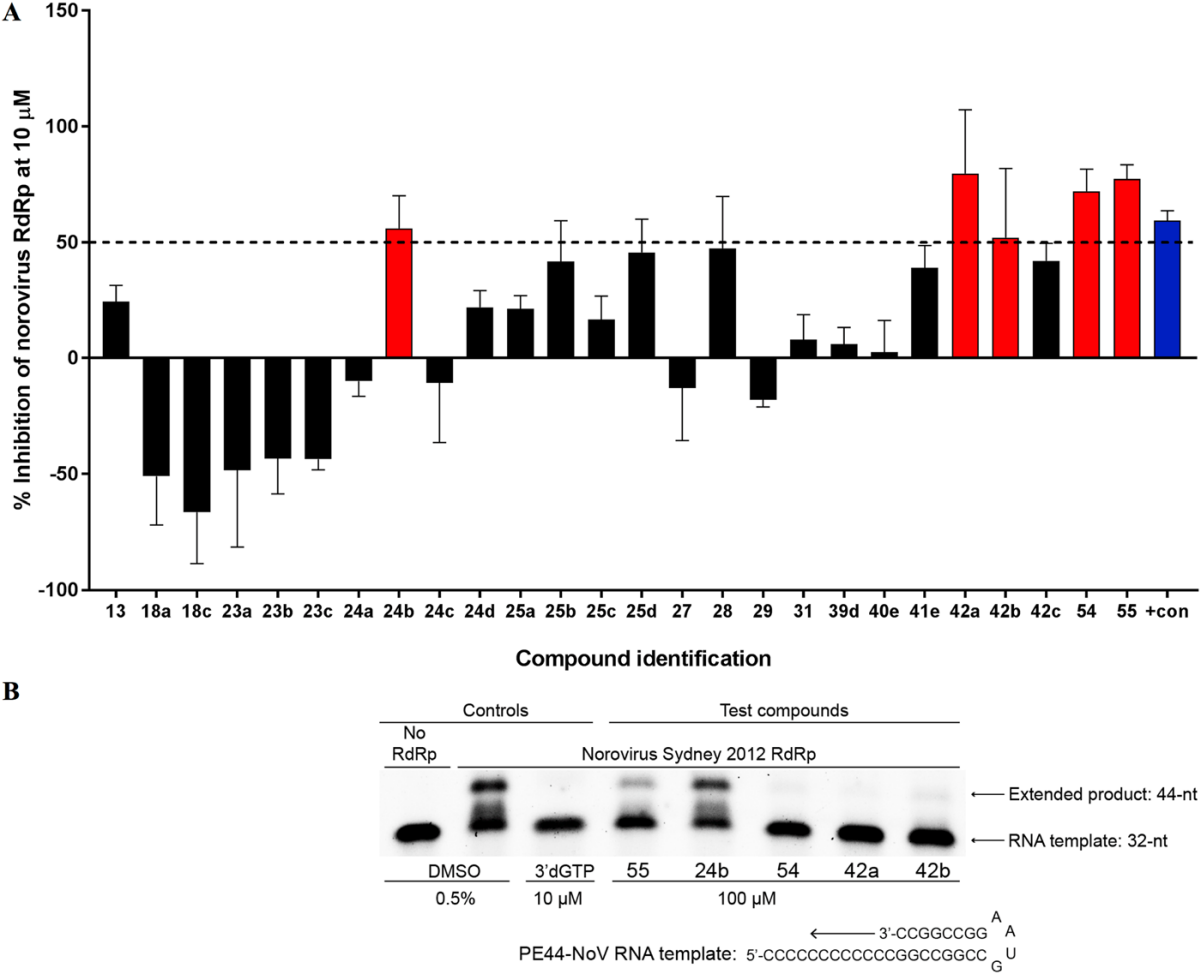
Secondary screen for inhibition of human norovirus Sydney 2012 RdRp activity. (**A**) The inhibitory effects of 26 compounds were examined against norovirus RdRp activity using a fluorescent activity assay at 10 µM. Five compounds that exhibited more than 50% inhibition (red bars) were chosen for further assessment with a counter-screen gel-shift RdRp assay. The positive control (blue bar) is the norovirus NNI RdRp inhibitor NIC02^14^. The mean values of triplicate datasets with standard deviations are shown. (**B**) A counter-screen-gel-shift assay was used to confirm norovirus RdRp inhibitory activity of the five compounds identified in panel (**A**). PE44-NoV RNA templates (32 nucleotides) were extended (44 nucleotides) by the RdRp in the absence of any test compounds (0.5% DMSO [vol/vol] negative control) or with test compounds at a fixed concentration of 100 µM. The nucleoside analogue 3′-deoxyguanosine-5′-triphosphate (3′dGTP) was used as a positive control (10 µM) to demonstrate complete inhibition, and no RdRp was used as a negative control. Full-length gels are presented in Supplementary Fig. S3.

Results of the assay are reported in Fig. 11A. The re-prepared **13** demonstrated a similar level of inhibition compared to the initial screening data (25% inhibition at 10 µM, cf. 37% inhibition at 10 µM for the commercial variant), further supporting the reproducibility of the assay and more importantly confirming the RdRp inhibitory activity associated with this particular molecule. The shorter phenylfuran-2-yl-(methylene)thiazolidine-2,4-dione derivatives (**18a, 18c, 27**), regardless of the substituent on the aromatic ring, and the furan-2-yl(methylene)-3-(morpholine-4-carbonyl)thiazolidine-2,4-dione (**31**) lacked RdRp inhibitory activity, indicating that RdRp inhibition was associated with the whole scaffold of **13** and not only with the methylbenzoic acid portion or the morpholine ring part. These derivatives are likely too small to entirely occupy the RdRp binding site area to block polymerase activity. The overall RdRp inhibitory activity of the derivatives of **13** seems to be the result of the combination of the two portions forming the molecule. In the morpholine derivatives, the most important influence for the activity was given by the free acid in position *para* of the aromatic ring. In fact, its removal (**23a**, **23c**) or its conversion to a methyl ester (**23b**, **39d**) lead to a complete loss of activity, whereas the derivative bearing only the free acid (**28**) showed improved activity when compared with the initial hit, inhibiting the RdRp activity by almost 50%. The methyl group at position *ortho* seems irrelevant for inhibitory activity, as demonstrated by the lack of activity of **23c**, and its removal, together with the presence of the free acid, lead to a significant increment of inhibitory activity. In the piperidine compounds, an unsubstituted aromatic ring (**24a**) and an *ortho* methyl substituent (**24c**) are related with the loss of inhibitory activity, giving the same result obtained for the morpholine series. Replacement of the morpholine ring with piperidine (**40e**) in the original structure of **13** abolished the activity, whereas esterification of the same derivative (**24d**) resulted in 25% inhibition, similar to the initial hit. Removal of the *ortho* methyl group of **24d** (**24b**) increased the degree of RdRp inhibitory activity up to 60%, compared with **13**, confirming the detrimental effect of the *ortho* methyl group. Replacing the morpholine ring with a non-cyclic secondary amine, such as the dimethyl amine, slightly improved the activity compared to the initial hit, but a maximum of 45% inhibition at 10 µM was reached. Also in this case, the presence of the acid or the ester seems fundamental for activity retention (**25a**, **25c**). Insertion of an aniline moiety, in replacement of the morpholine, had a positive impact on inhibitory activity, with derivative **42a**, characterised by the unsubstituted aromatic ring on one side and the aniline on the other side, reaching 75% RdRp inhibition at 10 µM.

Hit **11** derivatives, in which the original thiazole ring has been replaced by an unsubstituted phenyl ring, showed 75% RdRp inhibition, with no substantial differences between the methyl benzene and the unsubstituted phenyl derivative (**55** vs. **54**), and an activity comparable with the inhibition found in the initial screening for **11**.

Among the tested compounds, five (**24b, 42a, 42b, 54, 55**) demonstrated more than 50% inhibition of the human norovirus Sydney 2012 RdRp at 10 µM (Fig. 11A). To confirm RdRp inhibition, a gel-shift activity assay was again used to examine the five selected compounds at a fixed concentration of 100 µM (Fig. 11B) and three of the five compounds exhibited near complete abolishment of norovirus RdRp activity (**42a, 42b, 54**), whilst **55** only displayed minimal inhibition and **24b** had no appreciable effect.

### Four compounds have low micromolar IC_50_ values against human norovirus RdRp

The two initial hits identified from the virtual screening (**11** and **13**), and the three newly synthesised derivatives, which exhibited near complete abolishment of norovirus RdRp activity in the gel-based assay (**42a, 42b, 54**), were further assessed using a fluorescent RdRp assay to ascertain IC_50_ values (concentration range examined: 0.1–100 µM). Dose-dependent inhibitory response curves were used to establish IC_50_ values for the five test compounds (Table 2). While IC_50_ values could be attained for four of the five compounds tested, **42b** displayed significant solubility issues, even in the 100% DMSO solvent. Therefore, **42b** was too insoluble to attain a reliable confidence interval in the dose-response experiments and was not examined further. However, from the other four compounds, hit **11** and its phenyl derivative **54** gave the lowest IC_50_ values, 3–5 fold improved compared with **13** and its derivative **42a**, confirming increased RdRp inhibitory activity associated with this scaffold.

**Table 2:** IC_50_ values of hit compounds against human norovirus Sydney 2012 RdRp activities.

Compound Name	Structure	Molecular weight	IC_50_ values [µM]^A^ (95% confidence interval)
**11**		506.56	5.0 (2.4–10.6)
**13**		456.47	15.0 (7.4–27.4)
**54**		485.52	5.6 (4.1–7.6)
**42a**		404.45	27.4 (8.3–81.4)
**42b**		462.48	ND^B^

^A^The mean values of triplicate datasets are shown from at least two independent experiments. ^B^Compound too insoluble to attain an accurate IC_50_ measurement.

### Broad-spectrum calicivirus RdRp inhibitory activity

**Figure 12:**
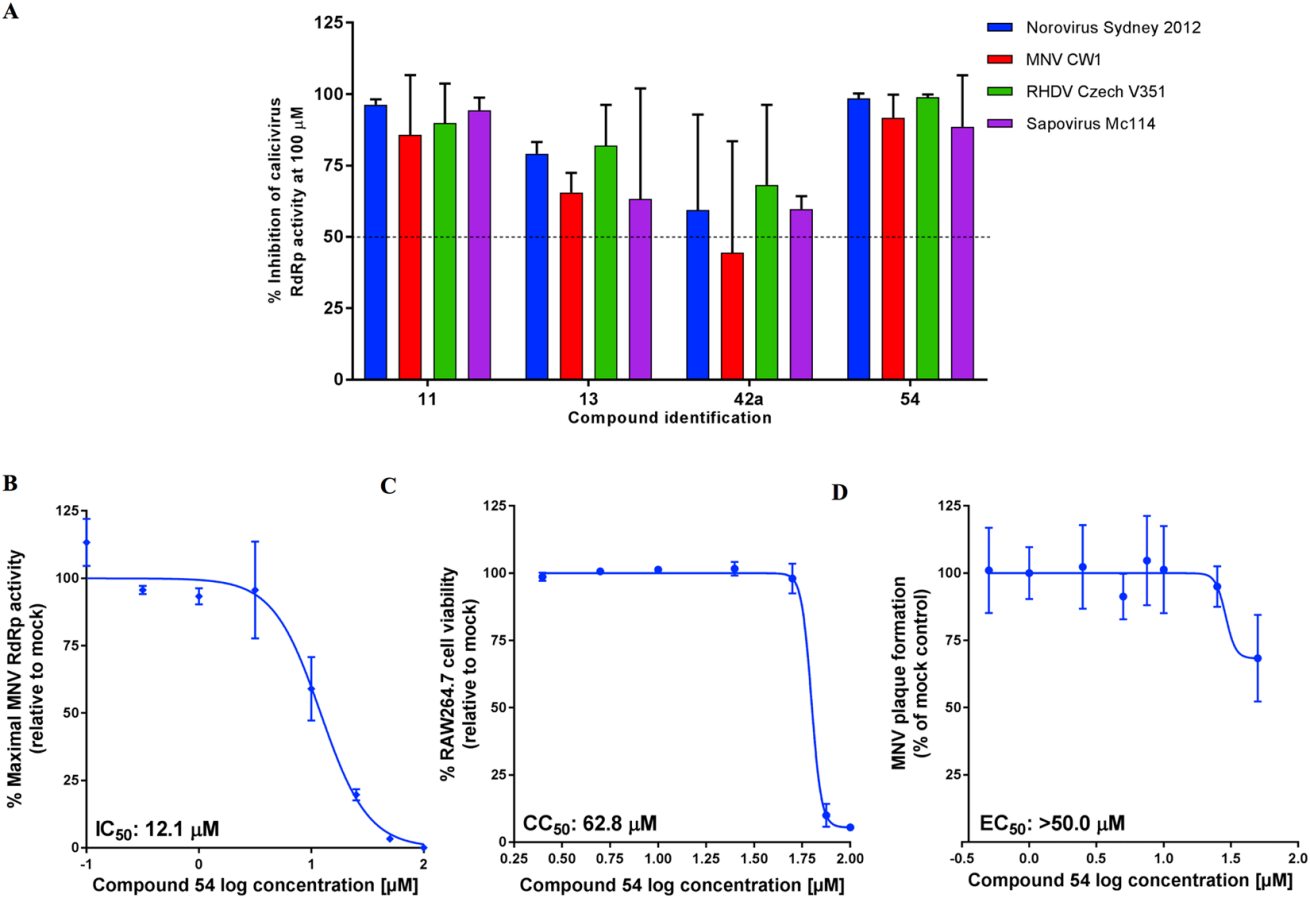
Broad-spectrum activity of hit compounds against calicivirus RdRps and examination of 54 in cell culture. (**A**) Effects of four compounds were evaluated against calicivirus RdRp activities (norovirus, MNV, RHDV and sapovirus) using an *in vitro* fluorescent activity assay at a fixed concentration of 100 µM. The mean values of triplicate datasets from at least two independent experiments are shown with standard deviations. (**B**) Dose-response effects of **54** (0.1–100 µM) were examined against MNV RdRp activity *in vitro*. The IC_50_ value and 95% confidence interval (CI) are shown on the graph. The mean values of triplicate datasets are shown with standard deviations. (**C**) RAW264.7 mouse macrophage cell viability was assessed with **54** (0.5–100 µM). The mean values of triplicate datasets from three independent experiments are shown with standard deviations. (**D**) The effects of **54** (0.5–50 µM) were assessed against MNV plaque formation in a plaque reduction assay. The mean values of triplicate datasets are shown with standard deviations.

The antiviral activity of the four compounds (**11**, **13**, **42a** and **54**) were examined for inhibition of RNA polymerases from other viruses spanning three genera of the *Caliciviridae* family, including *Norovirus* (human norovirus and MNV), *Lagovirus* (rabbit haemorrhagic disease virus [RHDV]) and *Sapovirus* (human sapovirus). All four compounds examined exhibited broad-spectrum inhibitory activity of the calicivirus RdRps at a fixed concentration of 100 µM, ranging between 44.4% inhibition (**42a** with MNV RdRp) through to 99.0% inhibition; (**54** with RHDV RdRp) (Fig. 12A). The protein sequence of the examined calicivirus polymerases has an identity range between 30.2–61.4%, and therefore the broad-spectrum activity of these compounds could be attributed to a common, highly-conserved binding pocket across the RdRps, as has been previously reported with broad-spectrum activity of other site-B binding NNIs^16^. Moreover, the ability of each compound to inhibit the different RdRps in a similar range could be further confirmation of targeting a well-conserved binding site of the *Caliciviridae* family.

To ensure the four hit compounds selectively inhibited calicivirus polymerase activity, they were further counter screened against a viral-like RdRp (bacteriophage Φ6) and an RNA-dependent DNA polymerase Moloney murine leukaemia virus reverse transcriptase (MMLV RT). None of the lead compounds reached 50% inhibition of the Φ6 and MMLV polymerases examined up to 100 μM (Supplementary Fig. S4). Overall, this demonstrated specificity for the calicivirus RdRps, in particular for the human Sydney 2012 RdRp (Fig. 12A and Supplementary Fig. S4).

### MNV RdRp activity and cell culture evaluation on 54

As mentioned above, the only robust cellular assay available for the evaluation of potential norovirus inhibitors is the closely related MNV assay. Out of the novel compounds examined (**54** and **42a**; Fig. 12A), **42a** demonstrated weak inhibition of MNV RdRp activity (<50% at 100 µM in Fig. 12A), which did not warrant further testing in cell culture. Evaluation of compounds **11** and **13** against MNV plaque formation in cell culture^58^ at two fixed concentrations of 10 and 100 µM did not give promising results. Compound **11** demonstrated 37.1% ± 2.7% inhibition of MNV plaque formation at 10 µM, but was cytotoxic at 100 µM, while compound **13** only reached 21.0% ± 1.2% inhibition of MNV plaque formation at 100 µM with no substantial differences between 10 and 100 µM (Supplementary Fig. S5). Compound **54**, a novel chemical entity, demonstrated the broadest spectra of antiviral activity across the four RdRps, and the highest level of inhibition of the MNV RdRp (Fig. 12A). Consequently, **54** was further assessed to determine the IC_50_ value against MNV RdRp activity using an *in vitro* fluorescent assay. **54** (0.1–100 µM) displayed an IC_50_ of 12.1 µM against MNV RdRp (Fig. 12B). **54** was then assessed for cytotoxicity against RAW264.7 mouse macrophage cells using a cell viability assay. **54** (0.5–100 µM) exhibited a mean half maximal cytotoxic concentration (CC_50_) of 62.8 µM (Fig. 12C). Using the plaque reduction assay, the inhibitory effects of **54** (0.5–50 µM) were examined against MNV plaque formation in cell culture. There was no appreciable effect on plaque numbers up to 25 µM, with RAW264.7 cells thinning at 50 µM, due to cytotoxicity (Fig. 12D). While **54** demonstrated an IC_50_ of 12.1 µM against the MNV RdRp *in vitro*, it had little effect against MNV in cell culture (EC_50_ > 50 µM). This could be due to poor cell membrane permeability of **54**, as observed with PPNDS^16^ and suramin^40^, which, despite inhibiting the RdRp in an isolated enzyme activity assay, demonstrated a lack of antiviral activity in the cell-based assay. Compound solubility and its cell-membrane permeability properties could be further improved with structure-activity relationship enhancement of the **54** scaffold to facilitate cellular uptake, and therefore to increase antiviral efficacy. Work on this aspect is ongoing, and it will be reported in due course.

### Binding site identification for 54: competitive assay, mutational analyses and molecular docking studies

**Figure 13:**
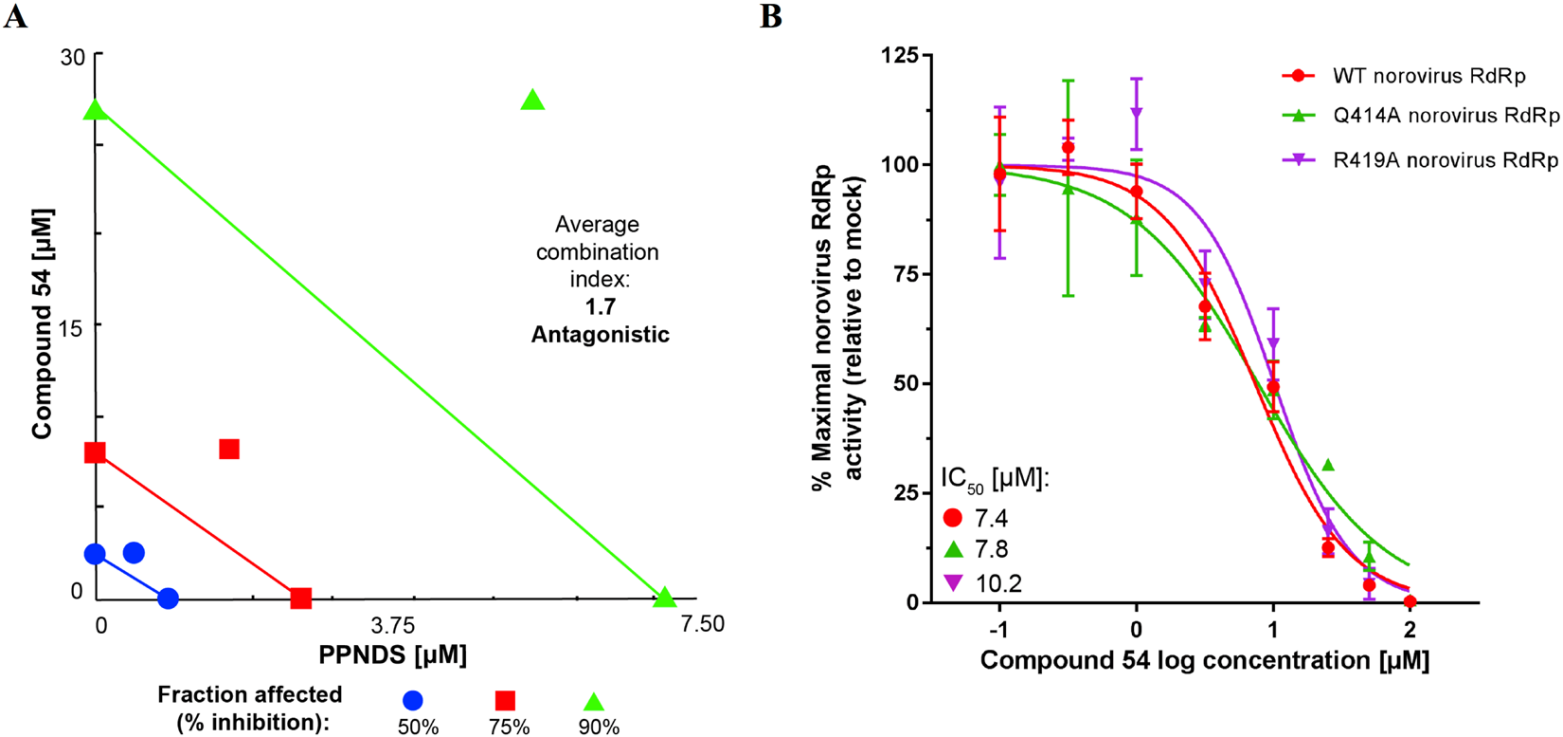
Compound 54 demonstrates competitive binding for the Site-B pocket with PPNDS, but displays unique amino acid interactions. (**A**) Isobologram of **54** (0.1–100 µM) and PPNDS (0.025–10 µM) examined in a 4:1 ratio respectively in combination. Together both molecules demonstrate antagonism against human norovirus Sydney 2012 RdRp activity with an average combination index of 1.7 over 50%, 75% and 90% inhibition. (**B**) Relative inhibitory effects of **54** on wild-type norovirus Sydney 2012 RdRp activity (red), mutants Q414 A (green) and R419A (purple), compared to mock treated samples (vehicle only). The IC_50_ values are shown on the graph. The mean values of triplicate datasets are shown from three independent experiments with standard deviations.

Despite the minimal activity in the cell culture system, RdRp inhibition by **54** is an important finding in the search of novel norovirus inhibitors. In order to improve the drug-like properties of the molecule without negatively influencing the RdRp inhibitory activity, and to design more potent derivatives, knowledge of the correct binding site of **54** is fundamental. As mentioned before, the broad-spectrum activity displayed by **54** could be attributed to a common, highly conserved binding pocket across the RdRps, that could be either site-A (suramin) or site-B (PPNDS). **54** and PPNDS were examined in a combinational study to test for antagonism (Fig. 13A), which would help demonstrate binding to the same RdRp region. PPNDS (0.025–25 µM) and **54** (0.1–100 µM) were examined alone and together in a 1:4 ratio respectively (Fig. 13A), and the average of the combination indices at 50% (1.6), 75% (1.7) and 90% (1.8) inhibition was 1.7. These results indicate antagonism, and therefore likely competitive binding of **54** and PPNDS within RdRp site-B. This is a relevant finding since the site-B pocket of the human norovirus RdRp has previously been shown to be an ideal antiviral target due to its highly conserved structure^16^.

Given the evidence of **54** being a site-B binding NNI, residues Q414 and R419 were mutated to alanine. These residues were previously shown by our research group and others to be critical for human norovirus RdRp site-B binding NNIs, and can create resistance to the drug through their mutagenesis^16,17,39^. After the mutant RdRps’ transcriptional activity was confirmed in a fluorescent RdRp assay (Q414A: 54%, R419A: 34% of wild-type activity, respectively), dose-response curves for **54** (0.1–100 µM) were produced to compare the IC_50_ values between the wild-type RdRp and the two mutant RdRps, Q414A and R419A (Fig. 13B). The mean IC_50_ value for the wild-type RdRp was 7.4 µM, which was similar to the IC_50_ values for Q414A RdRp (7.8 µM) and R419A RdRp (10.2 µM). The lack of effect of these two mutations on the inhibitory activity of **54**, together with the antagonism found with PPNDS, suggest that **54** may bind the site-B pocket of the polymerase with no specific interactions with Q414 and R419.

**Figure 14:**
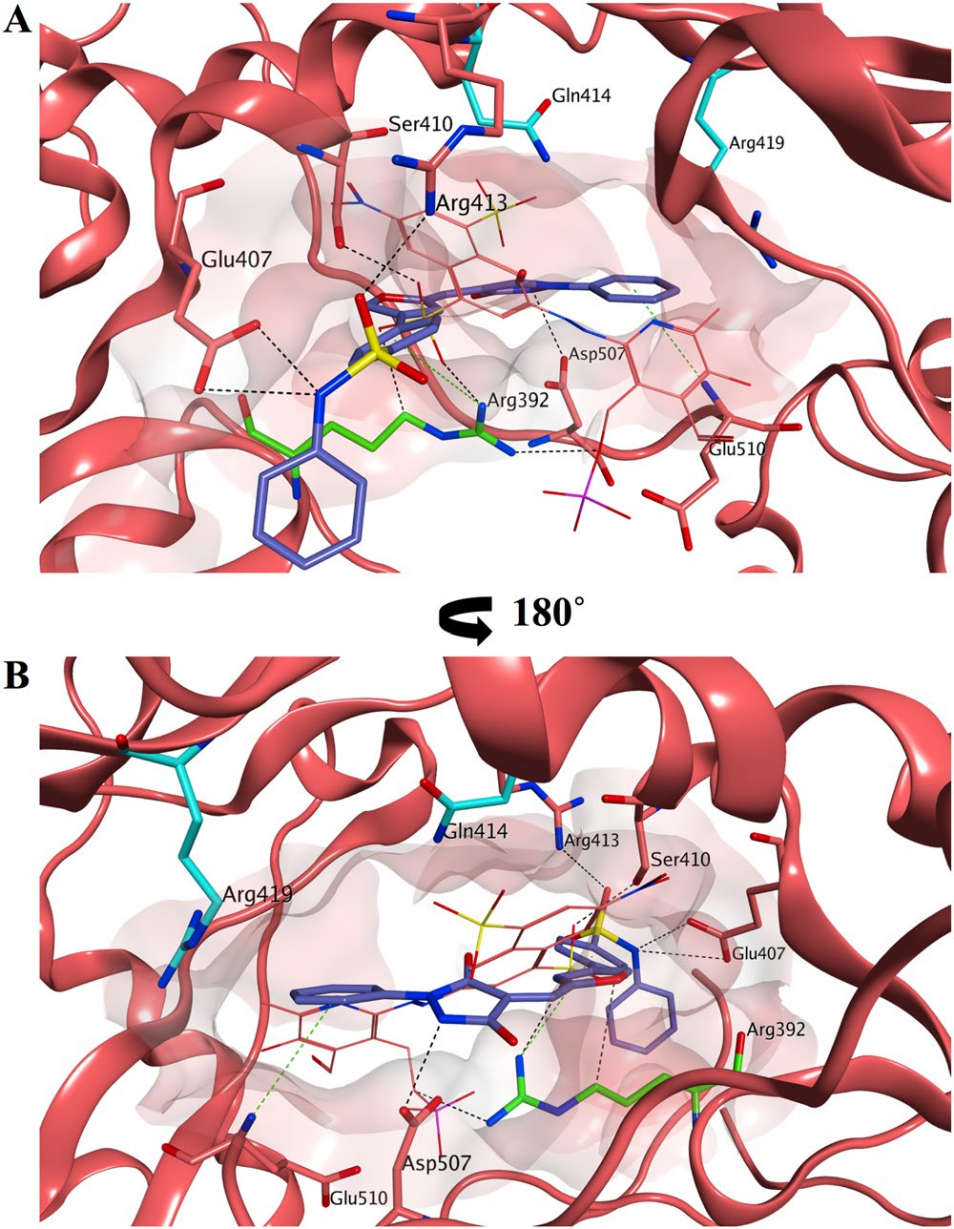
Proposed binding mode for 54 in the human norovirus RdRp. (**A**) Compound **54** (carbon atoms in lilac) is occupying site-B in a different orientation compared to PPNDS (carbon atoms in pink) with its phenyl-benzenesulfonamide portion pointing out from the site-B interacting through its sulphonamide group with Glu407 and Arg413 and through an arene-cation interaction between Arg392 and the benzene ring. (**B**) The binding site rotated by 180° shows the phenyl-pyrazolidine portion of **54** occupying the area where PPNDS places its naphthylazo portion, the core area of site-B, making interactions with the surrounding amino acids (Asp507, Glu510). No interactions are present between **54** and the two residues, Glu414 and Arg419. The binding site area is represented as molecular surface. Human norovirus RdRp is represented as salmon ribbon.

**Figure 15:**
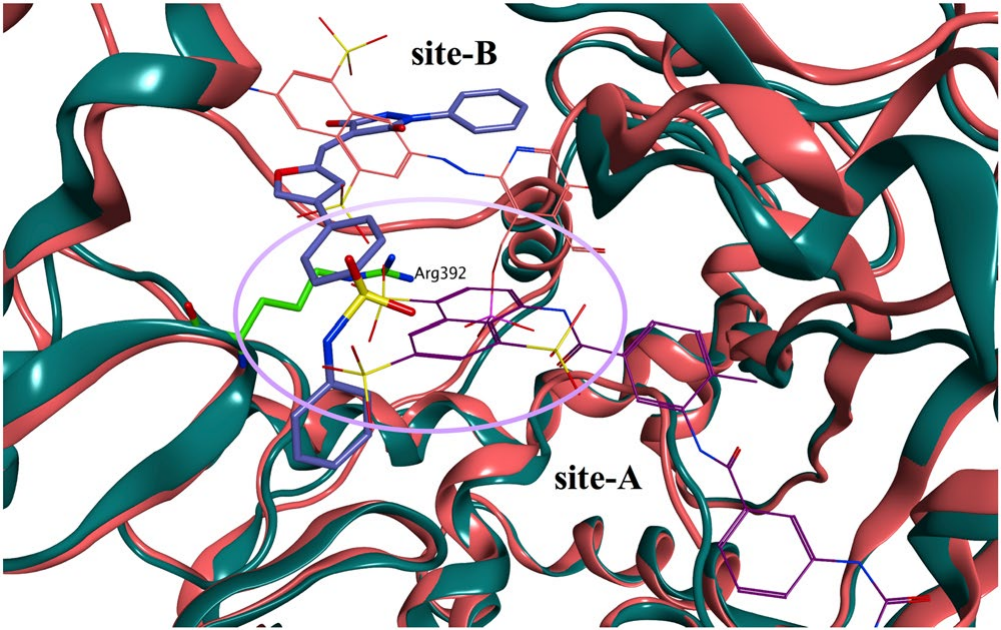
Overlap of site-A (suramin) and site-B (PPNDS) on the norovirus RdRp. Superposition of the MNV RdRp structure (dark green ribbon) in complex with suramin (carbon atoms in dark purple) and the human norovirus RdRp structure (salmon ribbon) in complex with PPNDS (carbon atoms in pink). **54** (carbon atoms in lilac) occupies the central core of site-B and extends up to the initial part of site-A (Arg392 area), where suramin places its sulphate head.

Molecular docking evaluations of **54** on the PPNDS binding site of human norovirus RdRp were performed using Glide SP. The proposed binding mode is shown in Fig. 14A,B. Compound **54** is predicted to occupy site-B in a different orientation compared to PPNDS, spanning an area that includes the central core of site-B and the initial part of site-A, where suramin places its sulphate head (Fig. 15). This binding could justify the antagonism results with PPNDS obtained in the combinational study. In fact, the value of 1.7 obtained is an indication of an antagonism/moderate-antagonism^59^, potentially meaning that **54** actually occupies only part of site-B to cause the antagonism, but does not occupy the entire site. The phenyl-benzenesulfonamide portion of **54** is pointing out from site-B toward the RdRp portion in which site-B and site-A overlap (Arg392 area), interacting through its sulphonamide group with Glu407 and Arg413, and through an arene-cation interaction between Arg392 and the benzene ring. No interactions are present between the phenyl ring and the protein, therefore its replacement by a thiazole, as in the initial hit **11**, does not seem to influence binding, in accordance with the similar inhibitory activity found for **54** and **11** in the biological assay. The phenyl-pyrazolidine portion is occupying the area where PPNDS places is naphthylazo part (Fig. 14B), within the core area of site-B, making interactions with the surrounding amino acids (Asp507, Glu510). The phenyl ring is perfectly fitted for this part of the binding site and the presence of substituents in *para* position could reduce the binding affinity for the site, causing steric clashes with the near residues, potentially justifying the minimal RdRp inhibition found for the methyl derivative **55**. Moreover, even if this portion of **54** is in proximity of Gln414 and Arg419, no interactions are present between the molecule and the two residues, in accordance with the lack of effect of the two mutations (Q414A and R419A) on the inhibitory activity of **54** (Fig. 13B).

## Conclusions and Future Work

A virtual screening evaluation of commercially available, drug-like compounds (~300,000) was performed on the suramin and PPNDS binding-sites of the human norovirus RdRp. These two RdRp inhibitors occupy two different sites, A and B, situated in the NTP access pathway located between the fingers and thumb domains, and overlap each other in the central part of the protein. Selected compounds (n = 62) were examined for inhibition of human norovirus RdRp activity using an *in vitro* transcription assay. Five candidates demonstrated dose-dependent RdRp inhibition (>25% inhibition at 10 µM and >50% inhibition at 100 µM), which was confirmed using a gel-shift RdRp assay. Among them, two compounds, **11** and **13**, were selected for an initial structure-activity relationship study. Synthetic efforts were made to develop the best synthetic pathway for the preparation of different derivatives of both hits. Among the newly prepared compounds, two showed interesting RdRp inhibition profiles. The two initial hits **11** and **13**, together with the two newly prepared derivatives **42a** and **54**, displayed IC_50_ values against human norovirus RdRp activity in the micromolar range (5.0–27.4 µM), with the best results obtained for **11** and its close derivative **54**. These four compounds also displayed broad-spectrum inhibitory activity of RdRps across three *Caliciviridae* genera, including *Norovirus*, *Lagovirus* and *Sapovirus*. There is little protein sequence identity between the examined calicivirus polymerases (30.2–61.4%), and therefore the broad-spectrum activity could be attributed to a common, highly-conserved binding pocket across the RdRps, as has been previously reported with broad-spectrum activity of other site-B binding NNIs^16^. While **54** demonstrated an IC_50_ of 12.1 µM against the MNV RdRp *in vitro*, it had little effect against MNV in cell culture (EC_50_ > 50 µM). This could be due to poor cell membrane permeability, as previously observed with PPNDS^16^ and suramin^40^.

Molecular docking studies revealed that the novel compound **54** is predicted to bind to the central core of site-B of the human norovirus RdRp, which has previously been shown to be an ideal antiviral target due to its highly conserved structure^16^. The proposed **54** binding mode was supported by combinational studies with PPNDS, the previously reported site-B binding norovirus NNI^17^. PPNDS and **54** were antagonistic in combination against norovirus RdRp activity, indicating that both compounds bind in the site-B pocket.

**54** may form distinct interactions with other residues within site-B, as mutations of previously reported site-B residues (Q414A and R419A) had no appreciable effect on the inhibitory activity of **54** compared to wild-type RdRp activity.

The mode of inhibitory action of PPNDS was reported to be through fixation of the C-terminus of the polymerase within the active site, blocking incoming NTPs and the RNA template^17^. It is possible that **54** works in the same fashion, and the actual binding site of **54** could be resolved through further crystallography and mutational studies.

In conclusion, a novel NNI scaffold, **54**, was found to be an effective and specific inhibitor of RdRps across three genera of the *Caliciviridae*, although there was little effect against MNV replication in cell culture, indicating that cell permeability could be further optimised. *In silico* and combinational studies suggest that **54** binds the highly conserved site-B in the RdRp, and therefore this compound represents a promising starting point for further structure-activity relationship enhancement, to improve potency, solubility and specificity profiles in the quest for an effective antiviral against human norovirus.

## Experimental

### Biology

#### Recombinant RdRp expression, purification and mutagenesis

Recombinant calicivirus RdRps (GenBank accession numbers) norovirus GII.Pe/GII.4 Sydney 2012 (KT239579), MNV GV CW1 (DQ285629), RHDV Czech V351 (KF594473.1), and SaV GI.1 Mc114 (AY237422) were expressed and purified as described previously^60,61^. RdRp resistance mutants were generated using the QuikChange site-directed mutagenesis kit (Agilent Technologies, Santa Clara, CA, USA) and confirmed by Sanger sequencing.

#### Quantitative RdRp activity and gel-based assays

Fluorescent RdRp activity assays were carried out as previously described^14,62^. Briefly, RdRp activity was quantified by monitoring the formation of double-stranded RNA (dsRNA) from a single stranded homopolymeric template, poly(C) (Sigma Aldrich, St Louis, MO, USA), using the fluorescent dye PicoGreen (Life Technologies, Carlsbad, CA, USA). RdRp assays were performed in 384-well plates, and each reaction mixture contained 400 ng enzyme, 45 µM GTP, 10 ng/µL poly(C) RNA, 2.5 mM MnCl_2_, 5 mM dithiothreitol, (DTT), 0.01% bovine serum albumin (BSA), and 0.005% Tween 20 in 20 mM Tris-HCl, pH 7.5, with a final reaction volume of 25 µL. The previous published norovirus NNI NIC02 was used as a positive control^14^. RdRps were incubated for 10 mins at 30 °C in the presence of the test compounds or the compound vehicle DMSO (0.5% vol/vol) before addition into the reaction mixture, which was then allowed to run 15 min at 30 °C then terminated with 10 mM EDTA, followed by PicoGreen staining and dsRNA quantitation. GraphPad Prism V6.05 (La Jolla, CA, USA) was used to plot the IC_50_ values. In cases where fluorescent enhancement was recorded for a compound instead of inhibition, a secondary gel-based polymerase activity assay was used as a counter-screen as described below, to exclude the possibility of RdRp activity enhancement (Supplementary Fig. S1). Primed elongation activity was examined in a gel-based assay as previously described^16,63^ using the RNA template (PE44-NoV, Fig. 4D). Gels were imaged using BioRad (Hercules, CA, U.SA) Geldoc Universal Hood II, running BioRad Image Lab software, V4.1, build 16. Antagonism was calculated using Compusyn software V1.0^59^. **54** and PPNDS (4:1 ratio) were tested alone and together against norovirus Sydney 2012 RdRp.

The *Pseudomonas* Φ6 bacteriophage RdRp (NEB) reactions were set up as described above (Fluorescent RdRp activity assays), using 400 ng of RdRp per reaction.

#### Cell culture of MNV and cytotoxicity assays

MNV-1 (CW1) culture assays were performed as previously described^14^. Replication was examined using plaque assays^58,64,65^. Cytotoxicity was examined using a CellTitre Blue (Promega, Madison, WI, USA) viability assay as described previously^14^.

#### Inhibition of viral reverse transcriptase

Lead hits were examined for inhibition of Moloney murine leukemia virus (MMLV) reverse transcriptase (RT) (kit M02535 (NEB)). MMLV RT activity reactions were carried out using 200 U MMLV RT (NEB) per reaction, with 250 ng homopolymeric poly(A) RNA template (average length 300 nucleotides, Sigma-Aldrich), 50 nM oligo dT_15_ primer, 1 × MMLV NTP buffer (NEB) and 0.1 mM dTTP in a final volume of 25 µL. The RT was incubated with the compound or the compound vehicle (0.5% DMSO) for 10 min, and then reactions were initiated by the addition of the RNA and primer mix. The reaction was incubated for 1 h at 42 °C then the RT was deactivated by incubation at 65° for 20 min. Picogreen stain was added and the relative fluorescence was measured as described above in the ‘Quantitative RdRp activity and gel-based assays’ section.

### Molecular Modelling

All molecular modelling studies were performed on a Viglen Genie Intel®Core^TM^ i7-3770 vPro CPU@ 3.40 GHz × 8 running Ubuntu 14.04. Molecular Operating Environment (MOE) 2015.10^66^, Maestro (Schrödinger Release 2017–1), LeadIT (version 2.1.8), OpenEye Scientific Software and PLANTS were used as molecular modelling software. Pharmacophoric filters were created within MOE choosing the PCH (polar-charged-hydrophobic) scheme. Shape-comparison screening was performed with ROCS version 3.2.1.4. Both shape and color screen criteria were applied to the query. Output conformations were ranked according to the Tanimoto combo score and the shape Tanimoto score. A library of commercially available compounds was downloaded from SPECS website (www.specs.net) in sdf format and prepared using the Conformational Search tool in MOE for the pharmacophore studies and the Omega version 2.5.1.4 for the shape-based screening.

RdRp crystal structures were downloaded from Protein Data Bank (PDB) (http://www.rcsb.org/); PDB code 3UR0 (suramin), 4LQ3 (PPNDS) and prepared with the MOE Protein Preparation tools for the pharmacophore studies and PLANTS docking. Docking PLANTS simulations for the virtual screening were performed using the following parameters: search algorithm: aco_ants 20, aco_evap 0.15, aco_sigma 2.0; binding site: bindingsite_center [−2.665 5.272 33.756] for suramine crystal [−19.229 −25.402 −4.634] for PPNDS crystal, bindingsite_radius 10; cluster algorithm: cluster_rmsd 2.0, cluster_strucures 5; scoring function: chemplp. The docking results obtained were then rescored using Glide XP and FlexX scoring functions.

Docking of **54** in the PPNDS binding site was performed using Glide docking program. The RdRp structure co-crystallised with PPNDS (PDB code: 4LQ3) was prepared using the Schrödinger Protein Preparation Wizard by assigning bond orders, adding hydrogens and performing a restrained energy minimization of the added hydrogens using the OPLS_2005 force field. An 11 Å docking grid (inner-box 11 Å and outer-box 220 Å) was prepared using as centroid the co-crystallised structure of PPNDS. Molecular docking of **54** was performed using Glide SP precision keeping the default parameters and setting 10 as number of output poses to include in the solution. The docking output database was saved as mol2 file and the docking poses visually inspected for their binding mode in MOE.

The visual inspection process, conducted as last step of the structure-based and the ligand-based virtual screening, was performed using MOE 2015.10. The docking poses of the compounds obtained from the *consensus* score procedure were evaluated considering the following criteria:

- ability of a compound to overall occupy the binding site (suramine or PPNDS binding site);
- number of interactions formed between the compound and the target protein (H-bonds, pie-pie interactions, etc.);
- coverage of different chemical scaffolds, discarding similar chemical entities;
- potential chemical instability;
- presence of potential toxic groups (multiple halogen atoms, aldehyde, etc.).

### Chemistry

All solvents and reagents were used as obtained from commercial sources unless otherwise indicated. All solvents used for chromatography were HPLC grade from Fisher Scientific (UK). All reactions were performed under a nitrogen atmosphere. ^1^H and ^13^C NMR spectra were recorded with a Bruker Avance DPX500 spectrometer operating at 500 MHz for ^1^H and 125 MHz for ^13^C, with Me_4_Si as internal standard. Deuterated chloroform was used as the solvent for NMR experiments, unless otherwise stated. ^1^H chemical shifts values (δ) are referenced to the residual non-deuterated components of the NMR solvents (δ = 7.26 ppm for CHCl_3_, etc.). The ^13^C chemical shifts (δ) are referenced to CDCl_3_ (central peak, δ = 77.0 ppm). Mass spectra were determined with a Bruker microTOF spectrometer using electrospray ionization (ESI source). For mass spectra, solutions were made in HPLC grade methanol. TLC was performed on silica gel 60 F254 plastic sheets. Flash column chromatography was performed using silica an Isolera Biotage system. UPLC-MS analysis was used for purity/mass determination of the tested compounds using a Waters UPLC system with both Diode Array detection and Electrospray (+’ve and –‘ve ion) MS detection. The stationary phase was a Waters Acquity UPLC BEH C18 1.7 um 2.1 × 50 mm column. The mobile phase was H_2_O containing 0.1% Formic acid (A) and MeCN containing 0.1% Formic acid (B). Column temperature: 40 °C. Sample diluent: acetonitrile. Sample concentration 1 µg/mL. Injection volume 2 µL. A linear gradient standard method (A) was used: 90% A (0.1 min), 90–0% A (2.6 min), 0% A (0.3 min), 90% A (0.1 min); flow rate 0.5 mL/min. All compounds tested in biological assays were >95% pure. Purity of intermediates was >90%, unless otherwise stated.

## Electronic supplementary material

Supplementary Information
