# Supplementary material for: In silico screening for human norovirus antivirals reveals a novel non-nucleoside inhibitor of the viral polymerase
Source: Sci Rep. 2018 Mar 7;8:4129. doi: 10.1038/s41598-018-22303-y (PMC5841303; doi:10.1038/s41598-018-22303-y)
Supplement: Supplementary file 1 — Supplementary Information [file 41598_2018_22303_MOESM1_ESM.pdf]

Supplementary Information

In silico screening for human norovirus antivirals reveals a novel non-nucleoside inhibitor of the viral polymerase

Salvatore Ferla*[1], Natalie E. Netzler*[2], Sebastiano Ferla[1,3], Sofia Veronese[1], Daniel Enosi Tuipulotu[2], Salvatore Guccione[3], Andrea Brancale[1], Peter A. White[2], Marcella Bassetto[1].

[1]*School of Pharmacy and Pharmaceutical Sciences, Cardiff University, Cardiff, United Kingdom;*

[2]*School of Biotechnology and Biomolecular Sciences, University of New South Wales, Sydney, NSW, Australia*

[3]*Dipartimento di Scienze del Farmaco, Università degli Studi di Catania, Italy*

* *These authors contributed equally to this work.*

Correspondence and requests for material s should be addressed to Dr. S. Ferla (email: ferlas1@cardiff.ac.uk).

Contents:

Pag S2 General method for the preparation of 16a-e (Method A)

Pag S4 General method for the preparation of 13, 18a-d, 27, 28, 30, 39d, 40e, 41e, 42a-c (Method B)

Pag S9 General method for the preparation of compounds 20-22, 34 (Method C)

Pag S10 General method for the preparation of compounds 23a-c, 24a-d, 25a-d, 29, 31, 33 (Method D)

Pag S16 General method for the preparation of compounds 36-38 (Method E)

Pag S16 Preparation of *4-Bromo-N-phenylbenzenesulfonamide*

Pag S17 General method for the preparation of compounds 46, 47 (Method F)

Pag S18 General method for the preparation of compounds 48, 49 (Method G)

Pag S18 Preparation of *4-(5-formylfuran-2-yl)-N-phenylbenzenesulfonamide* (53)

Pag S19 General method for the preparation of compounds 54, 55 (Method H)

Pag S20 Preparation of *4-(5-formylfuran-2-yl)benzoic acid* (26)

Pag S21, S22, S23 Figure S1, S2 and S3

Pag S24 Figure S4

Pag S25 Figure S5

Pag S26 References

General method for the preparation of 16a-e (Method A)

A solution of differently substituted phenylboronic acid (3 mmol), 5-bromofuran-2-carbaldehyde (2.86 mmol), $Pd(PPh_3)_4$ (0.1 mmol) and 2M aq. Na_2CO_3 (26 mL, 4.74 mmol) in THF (7.5 mL) was stirred at r.t. for 8h; stirring was continued at 60°C for additional 12h. The solution was cooled to r.t. and THF was removed under *vacuum*. The residue was partitioned between water (30 mL) and EtOAc (60mL). The organic layer was washed with brine (30 mL), dried over $MgSO_4$ and concentrated in *vacuum.* The crude residue was purified by

column chromatography giving the pure 5-arylfuran-2-carbaldehydes derivative. In some cases the pure product was obtained after the work-up and no further purification was needed.

5-Phenylfuran-2-carbaldehyde (16a) [1]

The data are in accordance with literature data. Purified by automated flash column chromatography (Biotage Isolera One, SNAP KP Sil 25g) eluting with *n*-hexane:EtOAc 100:0 v/v increasing to 80:20 v/v in 10 CV. Obtained in 90% yield as a yellow oil. ^{1}H-NMR ($CDCl_3$): δ 9.65 (s, 1H, C$\underline{H}$O), 7.83-7.82 (m, 2H), 7.46-7.43 (m, 2H), 7.41-7.38 (m, 1H), 7.32 (d, J = 3.7 Hz, 1H), 6.84 (d, J = 3.7 Hz, 1H).

Methyl 4-(5-formylfuran-2-yl)benzoate (16b) [1]

The data are in accordance with literature data. Product formation after stirring at r.t. for 8h. Purified by automated flash column chromatography (Biotage Isolera One, SNAP KP Sil 25g) eluting with *n*-hexane:EtOAc 100:0 v/v increasing to 40:60 v/v in 14 CV. Obtained in 98% yield as off-white solid. ^{1}H-NMR ($CDCl_3$): δ 9.66 (s, 1H), 8.07 (d, J = 8.3 Hz, 2H), 7.85 (d, J = 8.3 Hz, 2H), 7.33 (d, J = 3.7 Hz, 1H), 6.94 (d, J = 3.7 Hz, 1H), 3.92 (s, 3H).

5-(o-Tolyl)furan-2-carbaldehyde (16c) [2]

The data are in accordance with literature data. Purified by automated flash column chromatography (Biotage Isolera One, SNAP KP Sil 50g) eluting with *n*-hexane:EtOAc 100:0 v/v increasing to 80:20 v/v in 10 CV. Obtained in 62% yield as yellow solid. ^{1}H-NMR ($CDCl_3$): δ 9.70 (s, 1H), 7.86-7.82 (m, 1H), 7.38 (d, J = 3.7 Hz, 1H), 7.36-7.30 (m, 3H), 6.78 (d, J = 3.7 Hz, 1H), 2.58 (s, 3H).

Methyl 4-(5-formylfuran-2-yl)-3-methylbenzoate (16d)

Pure product formation after stirring at 60°C for 18h. Obtained in 83% yield as orange solid. ^{1}H-NMR ($CDCl_3$): δ 9.74 (s, 1H), 7.99-7.92 (m, 3H), 7.39 (d, J = 3.7 Hz, 1H), 6.89 (d, J = 3.7 Hz, 1H), 3.97 (s, 3H), 2.64 (s, 3H). ^{13}C-NMR ($CDCl_3$): δ 172.82, 161.83, 153.19, 147.40,

131.20, 129.45, 127.40, 127.32, 125.71, 124.55, 107.65, 47.53, 17.21. MS [ESI, m/z]: 245.0 $[M+H]^+$.

4-(5-Formylfuran-2-yl)-3-methylbenzoic acid (16e)

Pure product formation after stirring at 60˚C for 24h. Obtained in 68% yield as yellow solid. ^{1}H-NMR ($CDCl_3$): δ 13.00 (s, 1H), 9.68 (s, 1H), 7.93-7.92 (m, 1H), 7.91-7.89 (m, 2H), 7.70 (d, J = 3.7 Hz, 1H), 7.23 (d, J = 3.7 Hz, 1H), 2.59 (s, 3H). ^{13}C-NMR ($CDCl_3$): δ 183.53, 172.01, 161.97, 157.05, 141.02, 137.00, 136.74, 136.25, 134.02, 133.93, 118.40, 26.67. MS [ESI, m/z]: 231.0 $[M+H]^+$.

General method for the preparation of 13, 18a-d, 27, 28, 30, 39d, 40e, 41e, 42a-c (Method B)

A mixture of differently substituted furan-2-carbaldehyde derivatives (1 mmol), differently substituted thiazolidine-2,4-diones (2 mmol) and β-alanine (2 mmol) in glacial acetic acid (3 mL) was heated at 100°C for 1h. Upon completion of the reaction, the mixture was cooled and the reaction was quenched with water (10mL). The precipitate was filtered off and the solid was subsequently triturated with water, MeOH and *n*-hexane to afford the desired product.

(Z)-3-Methyl-4-(5-((3-(2-morpholino-2-oxoethyl)-2,4-dioxothiazolidin-5-ylidene)methyl)furan-2-yl)benzoic acid (13)

Purified by re-crystallization form EtOH. Obtained in 44% yield as brown solid. ^{1}H-NMR (DMSO-d_6): δ 13.04 (s, 1H), 7.97-7.91 (m, 3H), 7.87 (s, 1H), 7.38 (d, J = 3.7 Hz, 1H), 7.24 (d, J = 3.7 Hz, 1H), 4.62 (s, 2H), 3.68-3.63 (m, 2H), 361-3.56 (m, 2H), 3.56-3.53 (m, 2H), 3.47-3.42 (m, 2H), 2.61 (s, 3H). ^{13}C-NMR (DMSO-d_6): δ 168.10, 167.32, 165.49, 163.92, 156.27, 149.54, 135.63, 132.90, 132.26, 130.93, 127.93, 127.35, 122.29, 119.77, 118.47, 115.08, 66.40, 45.01, 42.99, 42.43, 22.30. Anal. Calcd for $C_{22}H_{20}N_2O_7S$: C, 57.89; H, 4.42;

N, 6.14. Found: C, 57.57; H, 4.74; N, 6.07. UPLC-MS: Rt 1.86 min, MS $(ESI)^+$ 457.2 $[M+H]^+$, λmax 396.3 nm.

(Z)-5-((5-Phenylfuran-2-yl)methylene)thiazolidine-2,4-dione (18a) [3]

The data are in accordance with literature data. Obtained in 65% yield as yellow solid. ^{1}H-NMR (DMSO-d_6): δ 12.45 (bs, 1H), 7.85-7.81 (m, 2H), 7.65 (s, 1H), 7.55-7.50 (m, 2H), 7.43-7.39 (m, 1H), 7.31-7.28 (m, 1H), 7.25-7.23 (m, 1H). ^{13}C-NMR (DMSO-d_6): δ 169.26, 167.61, 157.23, 149.32, 129.76, 129.44, 129.23, 124.66, 121.53, 118.38, 110.05. Anal. Calcd for $C_{14}H_9NO_3S$: C, 61.98; H, 3.34; N, 5.16. Found: C, 62.13; H, 3.60; N, 5.26. UPLC-MS: Rt 2.13, MS $(ESI)^+$ 271.99 $[M+H]^+$, λmax 396.3 nm.

Methyl (Z)-4-(5-((2,4-dioxothiazolidin-5-ylidene)methyl)furan-2-yl)benzoate (18b)

Obtained in 71% yield as orange solid. ^{1}H-NMR (DMSO-d_6): δ 12.52 (bs, 1H), 8.07 (d, J = 8.7 Hz, 2H), 7.92 (d, J = 8.7 Hz, 2H), 7.65 (s, 1H), 7.44 (d, J = 3.7 Hz, 1 H), 7.26 (d, J = 3.7 Hz, 1H), 3.88 (s, 3H).^{13}C-NMR (DMSO-d_6): δ 169.11, 167.51, 166.17, 155.83, 150.32, 133.22, 130.58, 129.59, 124.66, 121.78, 121.40, 118.13, 112.29, 52.73. MS [ESI, m/z]: 330.1 $[M+H]^+$.

(Z)-5-((5-(o-Tolyl)furan-2-yl)methylene)thiazolidine-2,4-dione (18c)

Obtained in 92% yield as yellow solid. ^{1}H-NMR (DMSO-d_6): δ 12.50 (bs, 1H), 7.77 (d, J = 7.6 Hz, 1H), 7.66 (s, 1H), 7.42-7.31 (m, 3H), 7.27 (d, J = 3.7 Hz, 1H), 7.04 (d, J = 3.7 Hz, 1H), 2.50 (s, 3H). ^{13}C-NMR (DMSO-d_6): δ 169.13, 167.61, 156.91, 148.88, 135.39, 132.05, 129.31, 128.65, 127.19, 127.04, 121.42, 120.54, 118.46, 113.33, 22.22. Anal. Calcd for $C_{15}H_{11}NO_3S$: C, 63.15; H, 3.89; N, 4.91. Found: C, 63.12; H, 4.21; N, 4.90. UPLC-MS: Rt 2.24 min, MS $(ESI)^+$ 285.9 $[M+H]^+$, λmax 389.1 nm.

Methyl (Z)-4-(5-((2,4-dioxothiazolidin-5-ylidene)methyl)furan-2-yl)-3-methylbenzoate (18d)

Obtained in 79% yield as orange solid. ^{1}H-NMR (DMSO-d_6): δ 12.54 (s, 1H), 7.98-7.89 (m, 3H), 7.68 (s, 1H), 7.30 (d, J = 3.7 Hz, 1H), 7.22 (d, J = 3.7 Hz, 1H), 3.88 (s, 3H), 2.59 (S,

3H). ^{13}C-NMR (DMSO-d_6): δ 166.03, 155.35, 149.62, 135.55, 132.69, 132.55, 129.29, 127.59, 126.82, 121.08, 120.96, 118.22, 117.69, 115.09, 114.96, 52.67, 22.19. MS [ESI, m/z]: 344.2 $[M+H]^+$.

(Z)-4-(5-((2,4-Dioxothiazolidin-5-ylidene)methyl)furan-2-yl)benzoic acid (27)

Obtained in 54% yield as brown solid. ^{1}H-NMR (DMSO-d_6): δ 13.07 (bs, 1H), 12.55 (s, 1H), 8.07 (d, J = 8.7 Hz, 2H), 7.92 (d, J = 8.7 Hz, 2H), 7.66 (s, 1H), 7.44 (d, J = 3.7 Hz, 1H), 7.27 (d, J = 3.7 Hz, 1 H). ^{13}C-NMR (DMSO-d_6): δ 169.17, 167.59, 167.26, 156.04, 150.22, 132.89, 130.88, 130.60, 124.57, 121.70, 121.42, 118.15, 112.05. Anal. Calcd for $C_{15}H_9NO_5S$: C, 57.14; H, 2.88; N, 4.44. Found: C, 56.80; H, 4.03; N, 4.41. UPLC-MS: Rt 1.73 min, MS $(ESI)^-$ 314.2 $[M-H]^-$, λmax 396.7 nm.

(Z)-4-(5-((3-(2-Morpholino-2-oxoethyl)-2,4-dioxothiazolidin-5-ylidene)methyl)furan-2-yl)benzoic acid (28)

Purified by re-crystallization form EtOH. Obtained in 26% yield as yellow solid. ^{1}H-NMR (DMSO-d_6): δ 13.14 (s, 1H), 8.09 (d, J = 8.7 Hz, 2H), 7.97 (d, J = 8.7 Hz, 2H), 7.86 (s, 1H), 7.48 (d, J = 3.7 Hz, 1H), 7.36 (d, J = 3.7 Hz, 1H), 4.63 (s, 2H), 3.67-3.63 (m, 2H), 3.61-3.57 (m, 2H), 3.56-3.54 (m, 2H), 3.47-3-43 (m, 2H). ^{13}C-NMR (DMSO-d_6): δ 168.27, 167.27, 165.51, 163.93, 156.54, 150.11, 132.75, 131.14, 130.78, 124.75, 122.41, 119.67, 118.65, 112.19, 66.40, 44.98, 42.97, 42.41. Anal. Calcd for $C_{21}H_{18}N_2O_7S$: C, 57.01; H, 4.10; N, 6.33. Found: C, 57.17; H, 4.05; N, 6.43. UPLC-MS: Rt 1.77 min, MS $(ESI)^+$ 443.2 $[M+H]^+$, λmax 401.2 nm.

(Z)-5-((5-Bromofuran-2-yl)methylene)thiazolidine-2,4-dione (30)

Obtained in 87% yield as brown solid. ^{1}H-NMR (DMSO-d_6): δ 12.54 (s, 1H), 7.55 (s, 1H), 7.11 (d, J = 3.7 Hz), 6.88 (d, J = 3.7 Hz, 1H). ^{13}C-NMR (DMSO-d_6): δ 168.81, 167.48, 151.80, 127.56, 121.70, 121.06, 117.69, 116.13. MS [ESI, m/z]: 272.9, 274.9 $[M+H]^+$.

Methyl-(Z)-3-methyl-4-(5-((3-(2-morpholino-2-oxoethyl)-2,4-dioxothiazolidin-5-ylidene)methyl)furan-2-yl)benzoate (39d)

Purified by automated flash column chromatography (Biotage Isolera One, SNAP KP Sil 10g) eluting with *n*-hexane:EtOAc 100:0 v/v increasing to 0:100 v/v in 13 CV. Obtained in 16% yield as orange solid. ^{1}H-NMR (DMSO-d$_6$): δ 7.98-7.95 (m, 1H), 7.94 (d, J = 3.7 Hz, 2H), 7.86 (s, 1H), 7.38 (d, J = 3.7 Hz, 1H), 7.24 (d, J = 3.7 Hz, 1H), 4.62 (s, 2H), 3.89 (s, 3H), 3.68-3.64 (m, 2H), 3.60-3.57 (m, 2H), 3.57-3.54 (m, 2H), 3.46-3.43 (m, 2H), 2.6 (s, 3H). ^{13}C-NMR (DMSO-d$_6$): δ 168.08, 166.25, 165.49, 163.92, 156.03, 149.65, 135.81, 132.69, 132.64, 129.63,127.80, 127.45, 122.26, 119.74, 118.63, 115.32, 66.40, 52.72, 43.00, 42.43, 40.68, 22.14. Anal. Calcd for $C_{23}H_{22}N_2O_7S$: C, 58.72; H, 4.71; N, 5.95. Found: C, 58.92; H, 5.18; N, 6.06. UPLC-MS: Rt 2.17 min, MS (ESI)$^+$ 471.2 [M+H]$^+$, λmax 395.1 nm.

(Z)-4-(5-((2,4-Dioxo-3-(2-oxo-2-(piperidin-1-yl)ethyl)thiazolidin-5-ylidene)methyl)furan-2-yl)-3-methylbenzoic acid (40e)

Obtained in 39% yield as yellow solid. ^{1}H-NMR (DMSO-d$_6$): δ 13.11 (s, 1H), 8.02-7.95 (m, 3H), 7.91 (s, 1H,), 7.43 (d, J = 3.7 Hz, 1H), 7.28 (d, J = 3.7 Hz, 1 H), 4.62 (s, 2H), 3.53 (t, 2H), 3.48 (t, 2H), 2.66 (s, 3H), 1.70-1.59 (m, 4H), 1.50 (s, 2H). ^{13}C-NMR (DMSO-d$_6$): δ 168.12, 167.32, 165.53, 163.19, 156.25, 149.55, 135.63, 132.90, 130.90, 127.93, 127.66, 127.35, 122.24, 119.71, 118.55, 115.09, 45.54, 43.12, 43.09, 26.33, 25.61, 24.25, 22.29. Anal. Calcd for $C_{23}H_{22}N_2O_6S$: C, 60.78; H, 4.88; N, 6.16. Found: C, 60.68; H, 4.70; N, 6.22. UPLC-MS: Rt 2.07 min, MS (ESI)$^+$ 455.2 [M+H]$^+$, λmax 396.3 nm.

(Z)-4-(5-((3-(2-(Dimethylamino)-2-oxoethyl)-2,4-dioxothiazolidin-5-ylidene)methyl)furan-2-yl)-3-methylbenzoic acid (41e)

Obtained in 88% yield as yellow solid. ^{1}H-NMR (DMSO-d$_6$): δ 13.03 (bs, 1H), 7.96-7.90 (m, 3H), 7.86 (s, 1H), 7.37 (d, J = 3.7 Hz, 1H), 7.23 (d, J = 3.7 Hz, 1H), 4.58 (s, 2H), 3.07 (s, 3H), 2.86 (s, 3H), 2.61 (s, 3H). ^{13}C-NMR (DMSO-d$_6$): δ 168.10, 167.33, 165.51, 164.86,

156.26, 149.54, 135.61, 132.90, 132.23, 131.00, 127.66, 127.44, 122.23, 119.72, 118.53, 115.06, 114.80, 43.15, 36.24, 35.70, 22.30. Anal. Calcd for $C_{20}H_{18}N_2O_6S$: C, 57.96; H, 4.38; N, 6.76. Found: C, 58.03; H, 4.63; N, 6.71. UPLC-MS: Rt 1.86 min, MS $(ESI)^+$ 415.3 $[M+H]^+$, λmax 395.1 nm.

(Z)-2-(2,4-Dioxo-5-((5-phenylfuran-2-yl)methylene)thiazolidin-3-yl)-N-phenylacetamide (42a)

Obtained in 99% yield as yellow solid. ^{1}H-NMR (DMSO-d_6): δ 10.39 (s, 1H,), 7.88-7.86 (m, 3H), 7.56 (m, 4H), 7.44 (m, 1H), 7.34 (m, 4H), 7.09 (t, J = 14.9 Hz, 1H), 4.52 (s, 2H). ^{13}C-NMR (DMSO-d_6): δ 165.54, 164.35, 157.78, 149.26, 138.90, 129.80, 129.63, 129.36, 122.83, 124.16, 122.55, 119.93, 119.63, 117.63, 110.25, 44.39. Anal. Calcd for $C_{22}H_{16}N_2O_4S$: C, 65.34; H, 3.99; N, 6.93. Found: C, 65.53; H, 4.20; N, 7.11 UPLC-MS: Rt 2.37 min, MS $(ESI)^+$ 405.2 $[M+H]^+$, λmax 398.7 nm.

Methyl-(Z)-4-(5-((2,4-dioxo-3-(2-oxo-2-(phenylamino)ethyl)thiazolidin-5-ylidene)methyl)furan-2-yl)benzoate (42b)

Obtained in 55% yield as yellow solid. ^{1}H-NMR (DMSO-d_6): δ 10.39 (s, 1H), 8.11 (d, J = 8.6 Hz, 2H), 7.99 (d , J = 8.6 Hz, 2H), 7.88 (s, 1H), 7.56 (d, J = 8.4 Hz, 2H), 7.50 (d, J = 6.2 Hz, 1H), 7.37-7.32 (m, 3H), 7.09 (m, 1H) 4.53 (s, 2H), 3.89 (s, 3H).^{13}C-NMR (DMSO-d_6): δ 168.27, 166.18, 165.50, 164.31, 156.35, 150.25, 138.90, 133.14, 130.65, 129.82, 129.36, 124.88, 124.17, 122.38, 119.71, 119.63, 118.84, 112.45, 52.75, 44.44. Anal. Calcd for $C_{24}H_{18}N_2O_6S$: C, 62.33; H, 3.92; N, 6.06. Found: C, 62.61; H, 4.13; N, 5.96. UPLC-MS: Rt 2.31 min, MS $(ESI)^+$ 463.1 $[M+H]^+$, λmax 398.7 nm.

(Z)-2-(2,4-Dioxo-5-((5-(o-tolyl)furan-2-yl)methylene)thiazolidin-3-yl)-N-phenylacetamide (42c)

Obtained in 55% yield as yellow solid. ^{1}H-NMR (DMSO-d_6): δ 10.39 (s, 1H), 7.88 (s, 1H), 7.80 (d, J = 8.7 Hz, 1H), 7.56 (d, J = 8.0 Hz, 2H), 7.44-7.32 (m, 6H), 7.10-7.07 (m, 2H) 4.52

(s, 2H), 2.54 (s, 3H). ^{13}C-NMR (DMSO-d_6): δ 168.25, 165.53, 157.53, 148.84, 138.90, 135.58, 132.09, 129.52, 129.35,128.61, 127.41, 127.10, 124.16, 122.44, 120.04, 119.63, 117.52, 113.51, 44.41, 22.17. Anal. Calcd for $C_{23}H_{18}N_2O_4S$: C, 66.02; H, 4.34; N, 6.69. Found: C, 64.91; H, 4.18; N, 6.87. UPLC-MS: Rt 2.45 min, MS $(ESI)^+$ 419.2 $[M+H]^+$, λmax 391.5 nm.

General method for the preparation of compounds 20-22, 34 (Method C)

Differently substituted amine (5.7 mmol) were suspended in DCM (10 mL) and the reaction mixture was cooled to 0°C in an ice-bath under nitrogen atmosphere. 2-Bromoacetyl chloride (0.48 mL, 5.7 mmol) was added dropwise and the solution was stirred at 0°C for 10 min. The reaction was then added dropwise (at 0°C) of DIPEA (1 mL, 5.7 mmol) and stirred at 0°C for 20 min. The reaction was stirred at r.t for 20 min. The reaction was added of saturated NH_4Cl (20 mL) solution and DCM (25 mL). The organic layer was washed with saturated aqueous $NaHCO_3$ and brine, then dried over $MgSO_4$ and concentrated under *vacuum* to afford the desired product.

2-Bromo-1-morpholinoethan-1-one (20) [4]

The data are in accordance with literature data. Obtained in 58% yield as brown oil. ^1H-NMR ($CDCl_3$): δ 3.87 (s, 2H), 3.78-3.64 (m, 6H), 3.56-3.53 (m, 2H).

*2-Bromo-1-(piperidin-1-yl)ethan-1-one (*21) [5]

The data are in accordance with literature data. Obtained in 62% yield as brown oil. ^1H-NMR ($CDCl_3$): δ 4.09 (s, 1H), 3.88 (s, 1H), 3.58 (t, 2H), 3.49-3.46 (m, 2H), 1.70-1.65 (m, 4H), 1.60 (s, 2H).

2-Bromo-N,N-dimethylacetamide (22) [6]

The data are in accordance with literature data. Obtained in 41% yield as brown oil. ^1H-NMR ($CDCl_3$): δ 4.10 (s, 2H), 3.12 (s, 3H), 3.01 (s, 3H).

2-Bromo-N-phenylacetamide (34) [7]

The data are in accordance with literature data. Obtained in 98% yield as light brown solid. ^{1}H-NMR ($CDCl_3$): δ **δ:** 8.24 (s, 1H), 7.54 (m, 2H), 7.37 (m, 2H,), 7.20-7.17 (m, 1H), 4.21 (s, 1H), 4.00 (s, 1H).

General method for the preparation of compounds 23a-c, 24a-d, 25a-d, 29, 31, 33 (Method D)

A solution of differently substituted thiazolidine-2,4-dione (1 mmol) in dry DMF (8.1 mL) was cooled to 0°C in an ice-bath under nitrogen atmosphere. Anhydrous K_2CO_3 (3 mmol) was added and the mixture was stirred at 0°C for 10 min. Differently substituted 2-bromoacetamide derivatives (1.1 mmol) were added and the mixture was stirred at r.t. overnight, then at 60°C for 72 h. When the reaction was completed, water was added and the reaction was extracted with EtOAc. The organic layer was dried over $MgSO_4$ and concentrated under *vacuum*. The crude reaction mixture was purified either by column chromatography or re-crystallization to obtain the pure desired product.

(Z)-3-(2-Morpholino-2-oxoethyl)-5-((5-phenylfuran-2-yl)methylene)thiazolidine-2,4-dione

(23a)

Purified by automated flash column chromatography (Biotage Isolera One, SNAP KP Sil 10g) eluting with *n*-hexane:EtOAc 100:0 v/v increasing to 0:100 v/v in 10 CV. Obtained in 46% yield as yellow solid. ^{1}H-NMR (DMSO-d_6): δ 7.86 (d, J = 7.4 Hz, 2H), 7.83 (s, 1H), 7.57-7.54 (m, 2H), 7.43 (t, J = 7.4 Hz, 1H), 7.34-7.31 (m, 2H), 4.62 (s, 2H), 3.66-3.63 (m, 2H), 3.59-3.58 (m, 2H), 3.56-3.54 (m, 2H), 3.46-3.42 (m, 2H). ^{13}C-NMR (DMSO-d_6): δ 168.35, 165.54, 163.96, 157.73, 149.24, 129.79, 129.62, 129.14, 124.81, 122.52, 119.85, 117.61, 110.23, 66.40, 49.07, 44.99, 42.92, 42.41. Anal. Calcd for $C_{20}H_{18}N_2O_5S$: C, 60.29; H, 4.55; N, 7.03. Found: C, 60.07; H, 4.78; N, 6.90. UPLC-MS: Rt 2.15 min, MS $(ESI)^+$ 399.3 $[M+H]^+$ 412.2 [M+Na]+, λmax 398.7 nm.

Methyl-(Z)-4-(5-((3-(2-morpholino-2-oxoethyl)-2,4-dioxothiazolidin-5-ylidene)methyl)furan-2-yl)benzoate (23b)

Purified by re-crystallization from MeOH. Obtained in 75% yield as orange solid. ^{1}H-NMR (DMSO-d$_6$): δ 8.10 (d, J = 8.6 Hz, 2H), 7.98 (d, J = 8.6 Hz, 2H), 7.87 (s, 1H), 7.51 (d, J = 3.7 Hz, 1H), 7.37 (d, J = 3.7 Hz, 1H), 4.65 (s, 2H), 3.90 (s, 3H), 3.66-3.62 (m, 2H), 3.60-3.53 (m, 4H), 3.46-3.42 (m, 2H). ^{13}C-NMR (DMSO-d$_6$): δ 168.25, 166.18, 165.50, 163.92, 156.30, 150.24, 133.14, 130.66, 129.78, 124.88, 122.39, 119.65, 118.81, 112.48, 66.40, 52.78, 44.98, 42.98, 42.41. Anal. Calcd for $C_{22}H_{20}N_2O_7S$: C, 57.89; H, 4.42; N, 6.14. Found: C, 58.03; H, 4.30; N, 6.24. UPLC-MS: Rt 2.08 min, MS (ESI)$^+$ 457.2 [M+H]$^+$ 479.1 [M+Na]+, λmax 399.9 nm.

(Z)-3-(2-Morpholino-2-oxoethyl)-5-((5-(o-tolyl)furan-2-yl)methylene)thiazolidine-2,4-dione (23c)

The mixture was stirred at r.t. for 6h, then at 60° C for 42h. The product, after extraction with EtOAc, was directly dried under *vacuum*. Purified by re-crystallization from MeOH/water. Obtained in 76% yield as yellow solid. ^{1}H-NMR (DMSO-d$_6$): δ 7.85 (s, 1H), 7.80 (d, J = 7.3 Hz, 1H), 7.44-7.33 (m, 4H), 7.09 (s, 1H), 4.62 (s, 2H), 3.64 (s, 2H), 3.56 (d, J = 17.3 Hz, 4H), 3.45 (s, 2H), 2.51 (s, 3H). ^{13}C-NMR (DMSO-d$_6$): δ 168.22, 165.53, 163.94, 157.45, 148.80, 135.55, 132.09, 129.50, 128.57, 127.56, 127.35, 122.44 119.96, 117.49, 113.51, 66.39, 44.98, 42.94, 42.40, 40.61, 22.21. Anal. Calcd for $C_{21}H_{20}N_2O_5S$: C, 61.15; H, 4.89; N, 6.79. Found: C, 61.34; H, 4.70; N, 6.92. UPLC-MS: Rt 2.24 min, MS (ESI)$^+$ 413.3 [M+H]$^+$, λmax 392.7 nm.

(Z)-3-(2-Oxo-2-(piperidin-1-yl)ethyl)-5-((5-phenylfuran-2-yl)methylene)thiazolidine-2,4-dione (24a)

The mixture was stirred at 60 °C for 20h. Purified by re-crystallization from MeOH/water. Obtained in 54% yield as orange solid. ^{1}H-NMR (DMSO-d$_6$): δ 7.87-7.85 (m, 2H), 7.82 (s,

1H), 7.55 (t, J = 8.0 Hz, 2H), 7.45-7.41 (m, 1H), 7.33-7.31 (m, 2H), 4.57 (s, 2H), 3.47 (t, J = 5.0 Hz, 2H), 3.43 (t, J = 5.0 Hz, 2H), 1.62-1.60 (m, 2H), 1.56-1.50 (m, 2H), 1.48-1.43 (m, 2H). ^{13}C-NMR (DMSO-d_6): δ 168.34, 165.57, 163.23, 157.72, 149.26, 129.79, 129.60, 129.16, 124.81, 122.44, 119.78, 117.70, 110.22, 45.54, 43.08, 43.05, 26.34, 25.61, 24.25. Anal. Calcd for $C_{21}H_{20}N_2O_4S$: C, 63.62; H, 5.08; N, 7.07. Found: C, 63.46; H, 4.96; N, 7.28. UPLC-MS: Rt 2.35 min, MS (ESI)$^+$ 397.3 [M+H]$^+$, λmax 397.5 nm.

Methyl-(Z)-4-(5-((2,4-dioxo-3-(2-oxo-2-(piperidin-1-yl)ethyl)thiazolidin-5-ylidene)methyl)furan-2-yl)benzoate (24b)

Purified by re-crystallization from MeOH/water. Obtained in 71% yield as yellow solid. ^{1}H-NMR (DMSO-d_6): δ 8.10 (d, J = 8.6 Hz, 2H), 7.97 (d, J = 8.6 Hz, 2H), 7.83 (s, 1H), 7.49 (d, J = 3.7 Hz, 1H), 7.34 (d, J = 3.7 Hz, 1H) 4.57 (s, 2H), 3.89 (s, 3H), 3.48 (s, 2H), 3.43 (s, 2H), 1.62-1.57 (m, 4H), 1.45 (s, 2H). ^{13}C-NMR (DMSO-d_6): δ 168.24, 163.19, 156.30, 150.25, 133.15, 130.63, 129.79, 124.85, 122.28, 119.56, 118.89, 112.43, 52.75, 45.54, 43.09, 26.34, 25.60, 24.25. Anal. Calcd for $C_{23}H_{22}N_2O_6S$: C, 60.78; H, 4.88; N, 6.16. Found: C, 60.92; H, 5.18; N, 6.22. UPLC-MS: Rt 2.30 min, MS (ESI)$^+$ 399.3 [M+H]$^+$ 412.2 [M+Na]+, λmax 406.0 nm.

(Z)-3-(2-oxo-2-(piperidin-1-yl)ethyl)-5-((5-(o-tolyl)furan-2-yl)methylene)thiazolidine-2,4-dione (24c)

Purified by re-crystallization from MeOH/water. Obtained in 64% yield as yellow solid. ^{1}H-NMR (DMSO-d_6): δ 7.84 (s, 1H), 7.80 (d, J = 7.7 Hz, 1H), 7.43-7.34 (m, 4H), 7.08 (d, J = 3.7 Hz, 1H), 4.56 (s, 2H), 3.47 (m, 2H), 3.42 (m, 2H), 2.54 (s, 3H), 1.64-1.52 (m, 4H), 1.49-1.41 (m, 2H). ^{13}C-NMR (DMSO-d_6): δ 168.22, 157.47, 148.83, 135.56, 132.08, 129.49, 126.61, 127.39, 127.08, 122.35, 119.90, 113.48, 45.54, 43.08, 43.05, 26.34, 25.61, 24.25, 22.17. Anal. Calcd for $C_{22}H_{22}N_2O_4S$: C, 64.37; H, 5.40; N, 6.82. Found: C, 64.31; H, 5.38; N, 7.01. UPLC-MS: Rt 2.44 min, MS (ESI)$^+$ 411.2 [M+H]$^+$, λmax 391.5 nm.

Methyl-(Z)-4-(5-((2,4-dioxo-3-(2-oxo-2-(piperidin-1-yl)ethyl)thiazolidin-5-ylidene)methyl)furan-2-yl)-3-methylbenzoate (24d)

The mixture was stirred at 60° C for 18h. Purified by re-crystallization from MeOH/water. Obtained in 57% yield as orange solid. ^{1}H-NMR (DMSO-d$_6$): δ 7.99-7.91 (m, 3H), 7.86 (s, 1H), 7.38 (d, J = 3.5 Hz, 1H), 7.26 (d, J = 3.5 Hz, 1H), 4.57 (s, 2H), 3.88 (s, 3H), 3.48 (s, 2H), 3.43 (s, 2H), 2.61 (s, 3H), 1.66-1.53 (m, 4H), 1.45 (s, 2H). ^{13}C-NMR (DMSO-d$_6$): δ 169.17, 167.35, 166.69, 164.08, 157.18, 150.79,136.99, 133.85, 133.72, 130.86, 129.04, 128.38, 123.30, 120.70, 119.78, 116.40, 53.98, 46.81, 44.20, 27.39, 26.61, 25.31, 23.48. Anal. Calcd for $C_{24}H_{24}N_2O_6S$: C, 61.53; H, 5.16; N, 5.98. Found: C, 61.39; H, 5.50; N, 5.95. UPLC-MS: Rt 2.37 min, MS (ESI)$^+$ 469.2 [M+H]$^+$, λmax 392.7 nm.

(Z)-2-(2,4-Dioxo-5-((5-phenylfuran-2-yl)methylene)thiazolidin-3-yl)-N,N-dimethylacetamide (25a)

Purified by re-crystallization from MeOH/water. Obtained in 42% yield as orange solid. ^{1}H-NMR (DMSO-d$_6$): δ 7.84 (m, 3H), 7.55 (m, 2H), 7.43 (m, J = 22.5 Hz, 1H), 7.32 (s, 2H), 4.57 (s, 2H), 3.07 (s, 3H), 2.86 (s, 3H).^{13}C-NMR (DMSO-d$_6$): δ 168.33, 165.56, 164.90, 157.72, 149.26, 129.76, 129.60, 129.17, 124.81, 122.43, 119.79, 117.71, 110.21, 43.08, 36.24, 35.70. Anal. Calcd for $C_{18}H_{16}N_2O_4S$: C, 60.66; H, 4.53; N, 7.86. Found: C, 60.81; H, 4.36; N, 7.94. UPLC-MS: Rt 2.15 min, MS (ESI)$^+$ 357.2 [M+H]$^+$, λmax 386.7 nm.

Methyl-(Z)-4-(5-((3-(2-(dimethylamino)-2-oxoethyl)-2,4-dioxothiazolidin-5-ylidene)methyl)furan-2-yl)benzoate (25b)

Purified by re-crystallization from MeOH/water. Obtained in 58% yield as orange solid. ^{1}H-NMR (DMSO-d$_6$): δ 8.10 (d, J = 8.7 Hz, 2H), 7.97 (d, J = 8.7 Hz, 2H), 7.84 (s, 1H), 7.49 (d, J = 3.7 Hz, 1H), 7.39 (d, J = 3.7 Hz, 1H) 4.58 (s, 2H), 3.89 (s, 3H), 3.07 (s, 3H), 2.86 (s, 3H). ^{13}C-NMR (DMSO-d$_6$): δ 168.22, 166.18, 165.51, 164.86, 156.29, 150.25, 133.15, 130.63, 129.79, 124.86, 122.28, 119.58, 118.90, 112.42, 52.75, 45.13, 36.25, 35.71. Anal. Calcd for

$C_{20}H_{18}N_2O_6S$: C, 57.96; H, 4.38; N, 6.76. Found: C, 57.73; H, 4.64; N, 6.71. UPLC-MS: Rt 2.10 min, MS $(ESI)^+$ 415.2 $[M+H]^+$, λmax 398.7 nm.

(Z)-2-(2,4-Dioxo-5-((5-(o-tolyl)furan-2-yl)methylene)thiazolidin-3-yl)-N,N-dimethylacetamide (25c)

Purified by re-crystallization from MeOH/water. Obtained in 46% yield as orange solid. ^{1}H-NMR (DMSO-d_6): δ 7.85 (s, 1H), 7.80 (d, J = 7.3 Hz, 1H), 7.43-7.35 (m, 4H), 7.09 (d, J = 7.3 Hz, 1H), 4.57 (s, 2H), 3.06 (s, 3H), 2.85 (s, 3H), 2.54 (s, 3H). ^{13}C-NMR (DMSO-d_6): δ 168.20, 165.55, 164.89, 157.47, 148.83, 135.56, 132.08, 129.46, 128.61, 127.39, 127.08, 122.34, 119.91, 117.59, 113.47, 43.10, 36.24, 35.70, 22.17. Anal. Calcd for $C_{19}H_{18}N_2O_4S$: C, 61.61; H, 4.90; N, 7.56. Found: C, 61.67; H, 4.91; N, 7.72. UPLC-MS: Rt 2.74 min, MS $(ESI)^+$ 371.3 $[M+H]^+$, λmax 392.7 nm.

Methyl-(Z)-4-(5-((3-(2-(dimethylamino)-2-oxoethyl)-2,4-dioxothiazolidin-5-ylidene)methyl)furan-2-yl)-3-methylbenzoate (25d)

The mixture was stirred at 60° C for 18h. Purified by re-crystallization from EtOH/water. Obtained in 47% yield as orange solid. ^{1}H-NMR (DMSO-d_6): δ 8.00-7.92 (m, 3H), 7.86 (s, 1H), 7.38 (d, J = 3.5 Hz, 1H), 7.26 (d, J = 3.5 Hz, 1H), 4.58 (s, 2H), 3.88 (s, 3H), 3.07 (s, 3H), 2.86 (s, 3H), 2.61 (s, 3H). ^{13}C-NMR (DMSO-d_6): δ 168.00, 166.04, 165.39, 164.61, 155.87, 149.49,135.68, 132.70, 132.30, 129.55, 127.73, 127.07, 122.14, 119.65, 118.48, 115.09, 52.67, 43.02, 36.12, 35.47, 22.06. Anal. Calcd for $C_{21}H_{20}N_2O_6S$: C, 58.85; H, 4.71; N, 6.54. Found: C, 58.71; H, 4.94; N, 6.49. UPLC-MS: Rt 2.18 min, MS $(ESI)^+$ 429.2 $[M+H]^+$ 412.2 [M+Na]+, λmax 393.9 nm.

2-Morpholino-2-oxoethyl-(Z)-4-(5-((2,4-dioxothiazolidin-5-ylidene)methyl)furan-2-yl)benzoate (29)

The mixture was stirred at 60°C for 48h. When the reaction was completed, 2M HCl was added (20 mL) and the reaction was extracted with EtOAc (25 mL). The organic layer was

dried over $MgSO_4$ and concentrated under *vacuum*. Purified by automated flash column chromatography (Biotage Isolera One, SNAP KP Sil 25g) eluting with DCM:MeOH 100:0 v/v increasing to 95:5 v/v in 15 CV. Obtained in 14% yield as orange solid. ^{1}H-NMR (DMSO-d$_6$): δ 12.56 (s, 1H), 8.12 (d, J = 8.7 Hz, 2H), 7.98 (d, J = 6.9 Hz, 2H), 7.66 (s, 1H), 7.49 (s, 1H), 7.27 (s, 1H), 5.10 (s, 2H), 3.64 (s, 2H), 3.59 (s, 2H), 3.46 (s, 6H). ^{13}C-NMR (DMSO-d$_6$): δ 165.31, 155.82, 150.40, 133.42, 130.82, 130.67, 129.42, 124.90, 124.73, 121.46, 118.15, 112.45, 66.47, 66.37, 62.54, 44.77, 42.09. Anal. Calcd for $C_{21}H_{18}N_2O_7S$: C, 57.01; H, 4.10; N, 6.33. Found: C, 57.2; H, 4.36; N, 6.42. UPLC-MS: Rt 1.78 min, MS (ESI)$^+$ 443.2 [M+H]$^+$, λmax 398.7 nm.

(Z)-5-((5-Bromofuran-2-yl)methylene)thiazolidine-2,4-dione (31)

The mixture was stirred at r.t. for 30h, then at 60° C for 64h. When the reaction was completed, 2M HCl was added (20 mL) and the reaction was extracted with EtOAc (25 mL). The product, which was precipitated, was collected by filtration and washed with EtOAc and *n*-hexane. Obtained in 54% yield as light brown solid. ^{1}H-NMR (DMSO-d$_6$): δ 7.85 (s, 1H), 7.80 (d, J = 7.3 Hz, 1H), 7.44-7.33 (m, 4H), 7.09 (s, 1H), 4.62 (s, 2H), 3.64 (s, 2H), 3.56 (d, J = 17.3 Hz, 4H), 3.45 (s, 2H), 2.51 (s, 3H). ^{13}C-NMR (DMSO-d$_6$): δ 168.22, 165.53, 163.94, 157.45, 148.80, 135.55, 132.09, 129.50, 128.57, 127.56, 127.35, 122.44 119.96, 117.49, 113.51, 66.39, 44.98, 42.94, 42.40, 40.61, 22.21. Anal. Calcd for $C_{14}H_{13}N_2O_5SBr$: C, 41.91; H, 3.27; N, 6.98. Found: C, 42.12; H, 3.54; N, 6.91. UPLC-MS: Rt 1.91min, MS (ESI)$^+$ 401.1 403.1 [M+H]$^+$, λmax 360.2 nm.

3-(2-Morpholino-2-oxoethyl)thiazolidine-2,4-dione (33)

The mixture was stirred at 80° C for 18h. The reaction was diluted with DCM (25 mL) and washed with water (20 mL). The organic phase was washed with brine, dried over $MgSO_4$ and concentrated under *vacuum*. Obtained in 33% yield as light pink solid. ^{1}H-NMR ($CDCl_3$): δ 4.43 (s, 2H), 4.08 (s, 2H), 3.78-3.76 (m, 2H), 3.74-3.72 (m, 2H), 3.64-3.62 (m,

2H), 3.51-3.49 (m, 2H). ^{13}C-NMR ($CDCl_3$): δ 171.65, 171.19, 163.01, 66.65, 66.20, 45.12, 42.50, 42.22, 33.96. MS [ESI, m/z]: 225.3 $[M+H]^+$.

General method for the preparation of compounds 36-38 (Method E)

To an ice-cooled and stirred solution of thiazolidine-2,4-dione (1 mmol) in THF (6.5 mL) was added NaH (1.1 mmol). The stirring was continued at room temperature until evolution of gas ceased. To the obtained solution was added differently substituted 2-bromoacetamide (1.5 mmol) at 0˚C and the mixture was refluxed for 20h. When the reaction was completed, THF was evaporated and the residue was partitioned between water (20mL) and EtOAc (25 mL). The organic layer was dried over $MgSO_4$ and concentrated under *vacuum*.

3-(2-Oxo-2-(piperidin-1-yl)ethyl)thiazolidine-2,4-dione (36)

Purified by re-precipitation from *n*-hexane/EtOAc. Obtained in 64% yield as brown solid. ^{1}H-NMR ($CDCl_3$): δ 4.43 (s, 2H), 4.06 (s, 2H), 3.56 (t, 2H), 3.42 (t, 2H), 1.72-1.65 (m, 4H), 1.61-1.57 (m, 2H). ^{13}C-NMR (DMSO-d_6): δ 171.68, 171.28, 162.41, 45.82, 43.48, 42.47, 33.91, 26.15, 25.25, 24.29. MS [ESI, m/z]: 243.3 $[M+H]^+$.

2-(2,4-Dioxothiazolidin-3-yl)-N,N-dimethylacetamide (37)

Obtained in 97% yield as brown solid. ^{1}H-NMR (DMSO-d_6): δ 4.39 (s, 2H), 4.06 (s, 2H), 3.05 (s, 3H), 2.95 (s, 3H). ^{13}C-NMR (DMSO-d_6): δ 172.39, 172.10, 164.85, 42.83, 36.19, 35.63, 34.33. MS [ESI, m/z]: 203.4$[M+H]^+$.

2-(2,4-Dioxothiazolidin-3-yl)-N-phenylacetamide (38) [8]

The data are in accordance with literature data. Purified by re-precipitation from *n*-hexane/EtOAc. Obtained in 48% yield as brown solid. ^{1}H-NMR (DMSO-d_6): δ 10.31 (s, 1H), 7.54 (d, J = 7.6 Hz, 2H), 7.32 (t, 2H), 7.08 (t, 1H), 4.33 (d, 4H).

Preparation of *4-Bromo-N-phenylbenzenesulfonamide* (52) [9]

4-bromobenzenesulfonyl chloride (4.88 mmol) dissolved in pyridine (1 mL/mmol) was added dropwise at 0°C to a stirring solution of aniline in anhydrous pyridine (0.4 mL/mmol). Once the addition was complete, the mixture was allowed to warm to r.t. and stirred overnight.

The reaction mixture was diluted with EtOAc (30 mL), washed with 2M HCl (20 mL) and brine (10mL). The organic phase was dried over $MgSO_4$ and concentrated under *vacuum*. The crude mixture was re-precipitate from *n*-hexane/EtOAc obtaining the pure desired product.

The data are in accordance with literature data. Purified by re-precipitation from *n*-hexane/EtOAc. Obtained in 64% yield as white solid. ^{1}H-NMR ($CDCl_3$): δ 7.66-7.62 (m, 2H), 7.61-7.58 (m, 2H), 7.30-7.27 (m, 2H), 7.20-7.16 (m, 1H), 7.10-7.07 (m, 2H), 6.53 (s, 1H).

General method for the preparation of compounds 46, 47 (Method F)

Differently substituted phenylhydrazine (1.5 mmol) was dissolved in anhydrous THF (1.1 mL/mmol) under nitrogen atmosphere and added of NEt_3 (1 mmol). The mixture was cooled to -10°C and a solution of ethyl 3-chloro-3-oxopropanoate (1 mmol) in anhydrous THF (4.4 mL/mmol eq) was added dropwise. The mixture was allowed to warm to r.t. and stirred for 3h. The reaction was concentrated under *vacuum*, extracted between EtOAc (30 mL) and water (20 mL), dried over $MgSO_4$ and concentrated under vacuum. The crude product was purified by flash column chromatography.

Ethyl 3-oxo-3-(2-phenylhydrazineyl)propanoate (46)

Purified by automated flash column chromatography (Biotage Isolera One, ZIP KP Sil 30g) eluting *n*-hexane/EtOAc 100:0 v/v increasing to 0:100 v/v in 13 CV. Obtained in 94% yield as yellow solid. ^{1}H-NMR ($CDCl_3$): δ 8.69 (s, 1H), 7.27-7.25 (m, 1H), 6.89 (d, J = 7.7 Hz,

2H), 6.85 (d, J = 7.7 Hz, 1H), 6.15 (d, J = 4.4 Hz, 1H), 4.31-4.26 (m, 2H), 4.19-4.11 (m, 1H), 3.47 (s, 2H), 1.36 (t, 3H). ^{13}C-NMR ($CDCl_3$): δ 168.83, 165.06, 147.61, 129.21, 121.44, 113.70, 62.02, 40.32, 14.08. MS [ESI, m/z]: 223.1 $[M+H]^+$.

Ethyl 3-oxo-3-(2-(p-tolyl)hydrazineyl)propanoate (47)

Purified by automated flash column chromatography (Biotage Isolera One, SNAP KP Sil 25g) eluting *n*-hexane/EtOAc 100:0 v/v increasing to 0:100 v/v in 13 CV. Obtained in 76% yield as yellow waxy solid. ^{1}H-NMR ($CDCl_3$): δ 88.68 (s, 1H), 7.10-7.03 (m, 2H), 6.79 (d, J = 8.2 Hz, 2H), 6.11 (d, J = 4.0 Hz), 4.29-4.23 (m, 2H), 3.44 (s, 2H), 2.28 (s, 3H), 1.35 (t, 3H). ^{13}C-NMR ($CDCl_3$): δ 168.83, 165.06, 163.26, 129.21, 121.44, 113.70, 62.02, 40.32, 20.87, 14.08. MS [ESI, m/z]: 237.1 $[M+H]^+$.

General method for the preparation of compounds 48, 49 (Method G)

Differently substituted ethyl 3-oxo-3-(2-phenylhydrazineyl)propanoate derivatives (1 mmol) were dissolved in EtOH (17 mL/mmol) and ethanolic 1M NaOH solution (3 mL/mmol) was added at r.t. The mixture was stirred for 40 min. Once the reaction was completed, 1M HCl (8 mL) and water was added. The reaction was then concentrated under *vacuum* to remove the alcohol and then extracted with EtOAc (4x30 mL) and water (20 mL), dried over $MgSO_4$ and concentrated under *vacuum* to afford the pure desired product.

1-Phenylpyrazolidine-3,5-dione (48) [10]

The data are in accordance with literature data. Obtained in 39% yield as yellow solid. ^{1}H-NMR ($CDCl_3$): δ 7.62-7.55 (m, 2H), 7.49-7.41 (m, 2H), 7.28-7.21 (m, 1H), 3.56-3.37 (m, 2H).

1-(p-Tolyl)pyrazolidine-3,5-dione (49)

Obtained in 67% yield as yellow solid. ^{1}H-NMR ($CDCl_3$): δ 7.44 (d, J = 8.0 Hz, 2H), 7.29 (s, 1H), 7.23 (s, 1H), 3.45 (s, 2H), 2.37 (s, 3H). ($CDCl_3$): δ 169.05, 163.26, 136.36, 132.83, 129.93, 119.28, 37.73, 20.97. MS [ESI, m/z]: 191.5 $[M+H]^+$.

Preparation of *4-(5-formylfuran-2-yl)-N-phenylbenzenesulfonamide* (53)

A solution of (4-formylcyclopenta-1,3-dien-1-yl)boronic acid (0.48 mmol), 4-bromo-*N*-phenylbenzenesulfonamide (0.48 mmol), K_3PO_4 and Pd(dppf) in a mixed solvent of water (0.17 mL) and DMF (0.88 mL) was heated in a microwave at 130°C for 75 min. When the reaction was completed, the mixture was diluted with DCM, washed with $NaHCO_3$, dried over $MgSO_4$ and concentrated under *vacuum*.

Purified by automated flash column chromatography (Biotage Isolera One, SNAP KP Sil 10g) eluting *n*-hexane:EtOAc 100:0 v/v increasing to 30:70 v/v in 13 CV. Obtained in 61% yield as yellow solid. ^{1}H-NMR ($CDCl_3$): δ 9.72 (s, 1H), 7.88-7.82 (m, 4H), 7.35 (d, J = 3.7 Hz, 1H), 7.30 (s, 1H), 7.27 (s, 1H), 7.19-7.15 (s, 1H), 7.13-7.10 (m, 2H), 6.96 (d, J = 3.7 Hz, 1H), 6.70 (s, 1H). ^{13}C-NMR ($CDCl_3$): δ 177.54, 136.04, 133.05, 129.49, 127.97, 127.89, 125.92, 125.75, 125.55, 122.13, 121.87, 109.92. MS [ESI, m/z]: 328.6 $[M+Na]^+$.

General method for the preparation of compounds 54, 55 (Method H)

4-(5-Formylfuran-2-yl)-*N*-phenylbenzenesulfonamide (1 mmol) and differently substituted 1-phenylpyrazolidine-3,5-dionse (1 mmol) were suspended in AcOH (13 mL/mmol) and the mixture was stirred at 120°C for 3h. Upon completion of the reaction, the mixture was cooled, quenched with water and the precipitate was filtered off. The solid was washed with water and *n*-hexane giving the pure desired product.

(E)-4-(5-((3,5-Dioxo-1-phenylpyrazolidin-4-ylidene)methyl)furan-2-yl)-N-phenylbenzenesulfonamide (54)

Obtained in 50% yield as brown solid. ^{1}H-NMR (DMSO-d_6): δ 11.26 (s, 1H), 10.35 (s, 1H), 8.56-8.38 (m, 1H), 8.14-8.09 (m, 2H), 7.89-7.84 (m, 2H), 7.82-7.64 (m, 3H), 7.59-7.55 (m, 1H), 7.49-7.44 (m, 2H), 7.28-7.19 (m, 3H), 7.12 (d, J = 7.9 Hz), 7.05 (t, 1H). ^{13}C-NMR (DMSO-d_6): δ 157.87, 150.83, 140.25, 137.88, 132.61, 129.70, 129.44, 129.37, 128.10, 128.08, 126.03, 124.89, 120.96, 120.92, 118.82, 115.49, 113.78, 113.74. Anal. Calcd for $C_{26}H_{19}N_3O_5S$: C, 64.32; H, 3.94; N, 8.65. Found: C, 64.27; H, 4.12; N, 8.61. UPLC-MS: Rt 2.15 min, MS (ESI)$^+$ 486.1 [M+H]$^+$, λmax 421.7 nm.

(E)-4-(5-((3,5-Dioxo-1-(p-tolyl)pyrazolidin-4-ylidene)methyl)furan-2-yl)-N-phenylbenzenesulfonamide (55)

Obtained in 17% yield as brown solid. ^{1}H-NMR (DMSO-d_6): δ 11.26 (s, 1H), 10.35 (s, 1H), 8.50 (s, 1H), 8.1 (t, 2H), 7.86 (d, J = 9 Hz, 2H), 7.73-7.59 (m, 3H), 7.56 (s, 1H), 7.30-7.22 (m, 4H), 7.12 (d, J = 7.9 Hz, 2H), 7.09-7.00 (m, 1H), 2.32 (s, 3H). ^{13}C-NMR (DMSO-d_6): δ 157.75, 157.68, 140.21, 137.89, 134.50, 132.62, 129.74, 129.70, 128.09, 128.07, 126.00, 124.87, 120.94, 120.91, 119.20, 118.83, 115.80, 115.64, 113.73, 113.69, 20.95. Anal. Calcd for $C_{27}H_{21}N_3O_5S$: C, 64.92; H, 4.24; N, 8.41. Found: C, 64.80; H, 4.50; N, 8.29. UPLC-MS: Rt 2.23 min, MS (ESI)$^+$ 500.2 [M+H]$^+$, λmax 421.7 nm.

Preparation of *4-(5-formylfuran-2-yl)benzoic acid* (26) [11]

A mixture of methyl 4-(5-formylfuran-2-yl)benzoate (0.48 mmol) and Na_2CO_3 (2.4 mmol) in water (15 mL) was heated at 100°C for 2 h until a clean solution was obtained. The solution was cooled and carefully acidified with 6M HCl solution. The precipitate was collected by filtration and washed with water and *n*-hexane.

The data are in accordance with literature data. Obtained in 99% yield as brown solid. ^{1}H-NMR ($CDCl_3$): δ 13.13 (bs, 1H), 9.67 (s, 1H), 8.05 (d, J = 8.7 Hz, 2H), 8.00 (d, J = 8.7 Hz, 2H), 7.70 (d, J = 3.7 Hz, 1H), 7.46 (d, J = 3.7 Hz, 1H).

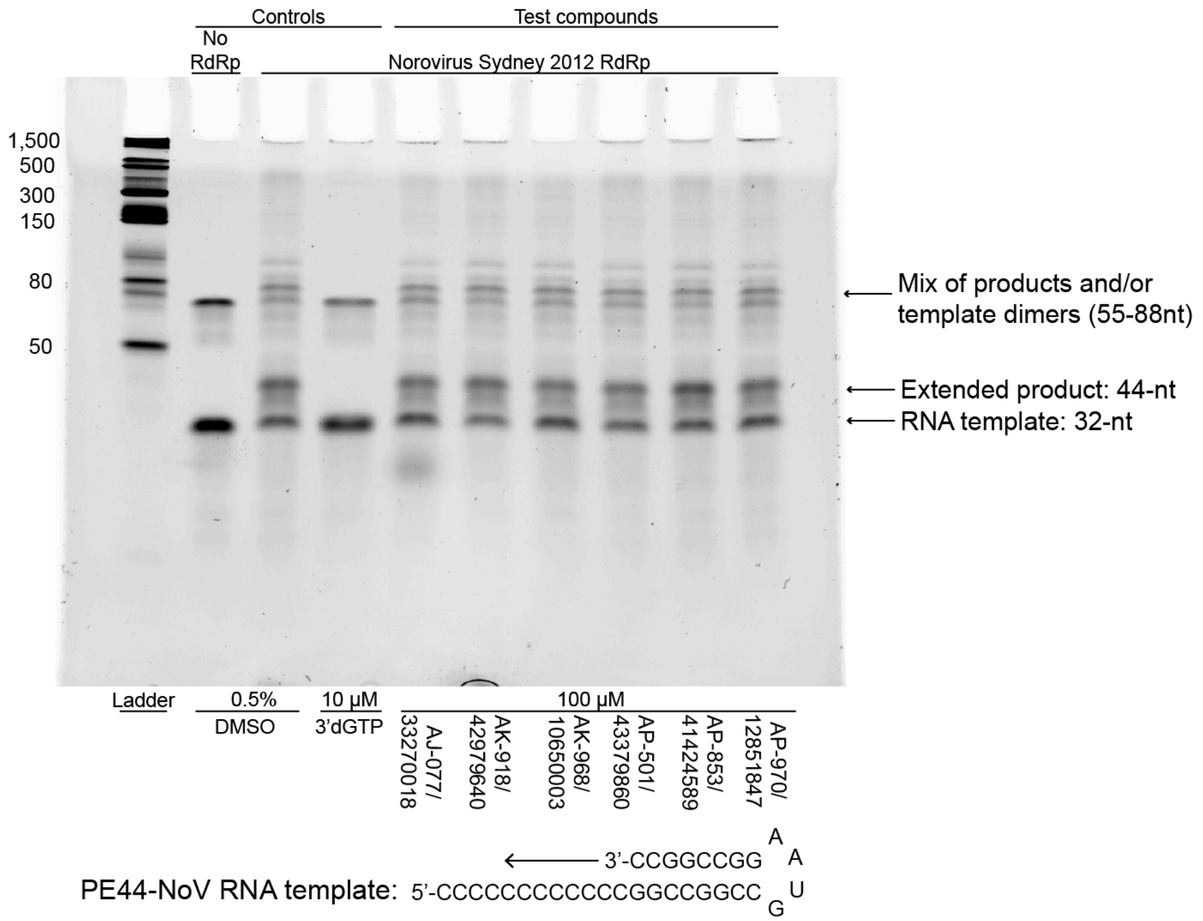

Supplementary Figure S1. Counter-screen for norovirus RdRp activity enhancement. A counter-screen gel-shift assay was used to check norovirus RdRp enhancement activity for six compounds that demonstrated greater than -75% inhibition in Figure 4B at 100 µM. PE44-NoV RNA templates of 32 nucleotides were extended to 44 nucleotides by the RdRp in the presence of test compounds at a fixed concentration of 100 µM, and in the absence of any compounds (0.5% DMSO [vol/vol], negative control). The nucleoside analogue 3'-deoxyguanosine-5'-triphosphate (3'dGTP) was used as a positive control (10 µM) to demonstrate complete inhibition of template elongation. Reagents containing no RdRp was used as a negative control.

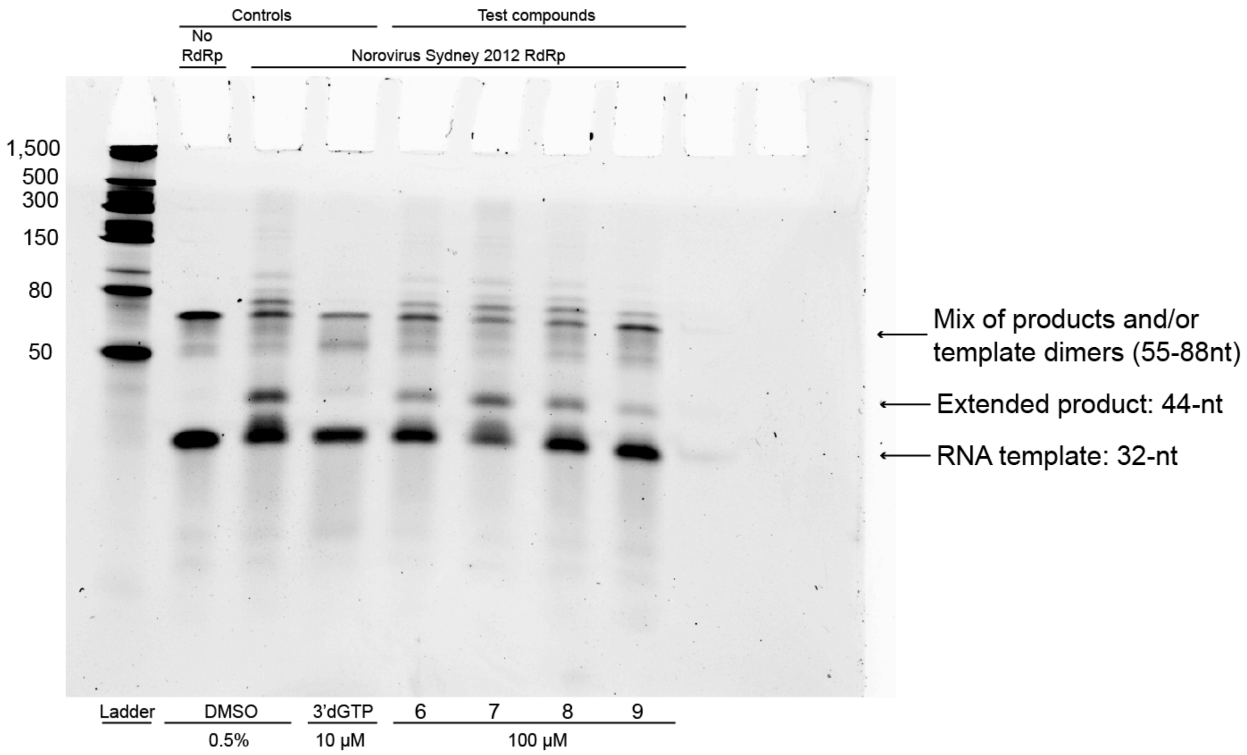

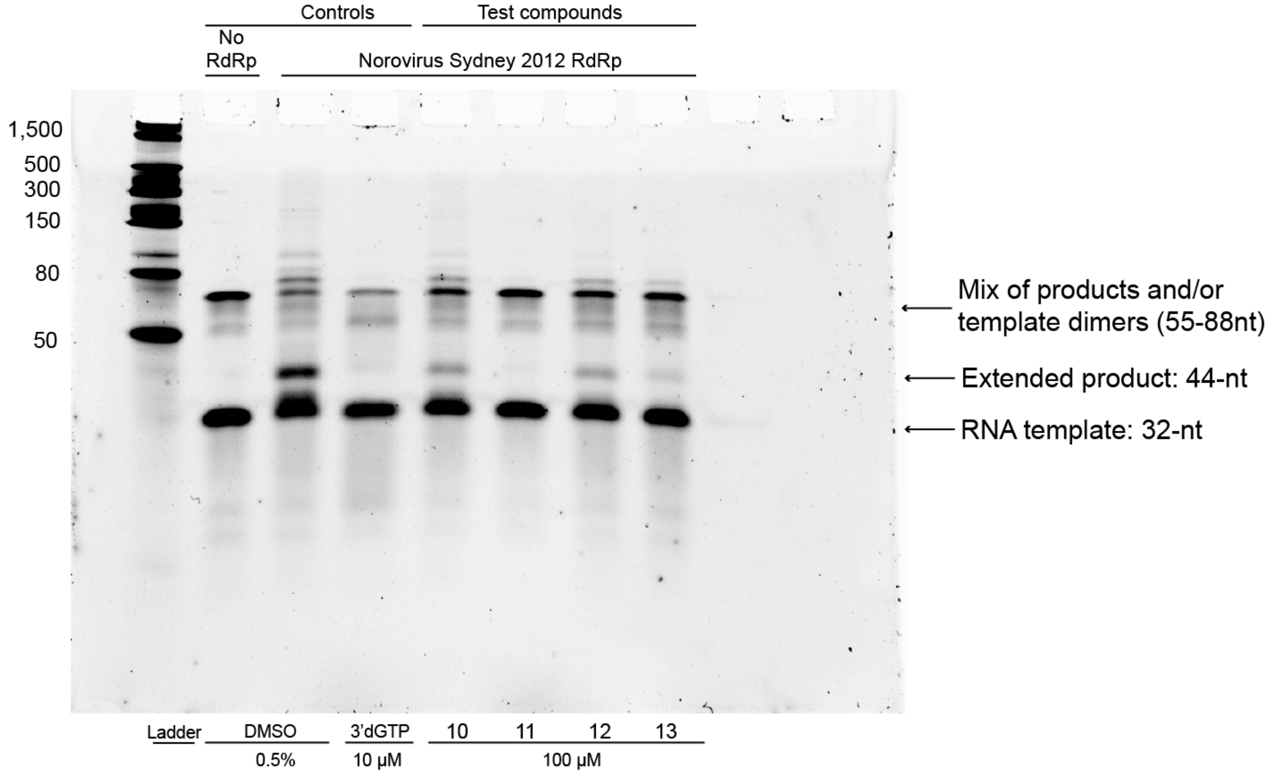

Supplementary Figure S2. Full length gel from Figure 4D. A counter-screen gel-shift assay was used to confirm norovirus RdRp inhibitory activity of the eight compounds identified in Figure 4A and 4B. The eight compounds identified in Figure 4 A and B were examined for inhibition of primed elongation activity. PE44-NoV RNA templates (32

nucleotides) were extended (44 nucleotides) by the RdRp in the absence of any test compounds (0.5% DMSO [vol/vol] negative control) or with test compounds at a fixed concentration of 100 μM. Lane 1 of each gel: Low range ssRNA ladder (NEB). Lane 2: No RdRp with DMSO (0.5% [vol/vol]), used as a negative control. Lane 3: Norovirus RdRp with DMSO (0.5% [vol/vol]), used as a positive control. Lane 4: Nucleoside analogue 3'-deoxyguanosine-5'-triphosphate (3'dGTP) was used as a positive control (10 μM) to demonstrate complete inhibition.

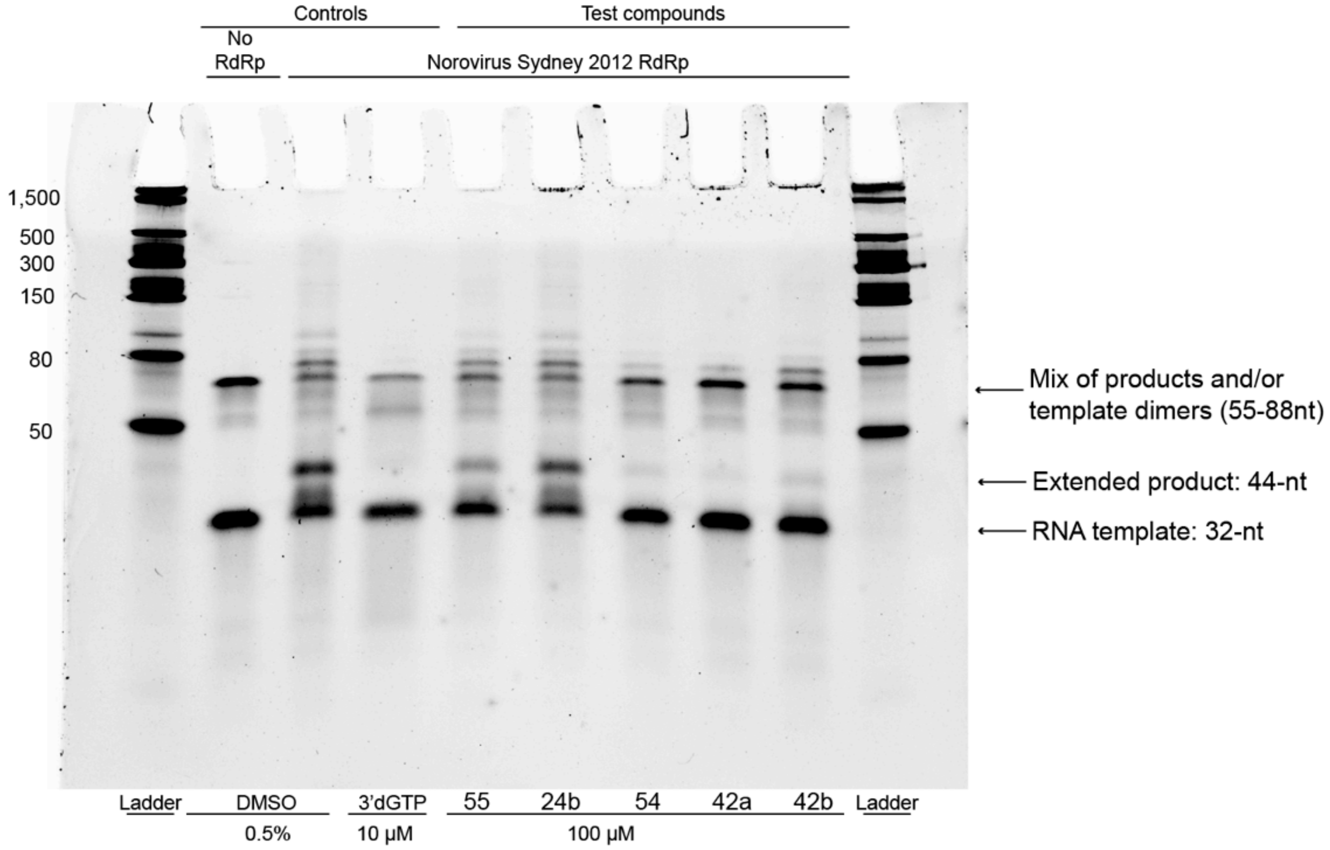

Supplementary Figure S3. Full length gel from Figure 11B. A counter-screen gel-shift assay was used to confirm norovirus RdRp inhibitory activity of the five compounds identified in Figure 11B. PE44-NoV RNA templates (32 nucleotides) were extended (44 nucleotides) by the RdRp in the absence of any test compounds (0.5% DMSO [vol/vol] negative control) or with test compounds at a fixed concentration of 100 μM. The nucleoside

analogue 3'-deoxyguanosine-5'-triphosphate (3'dGTP) was used as a positive control (10 μM) to demonstrate complete inhibition, and no RdRp was used as a negative control.

A)

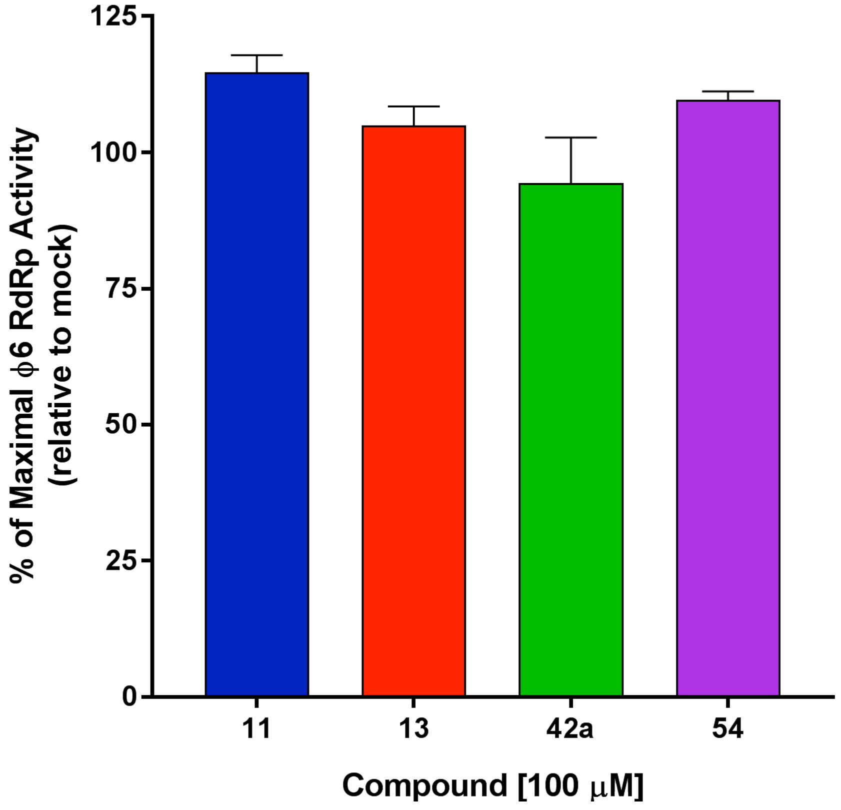

B)

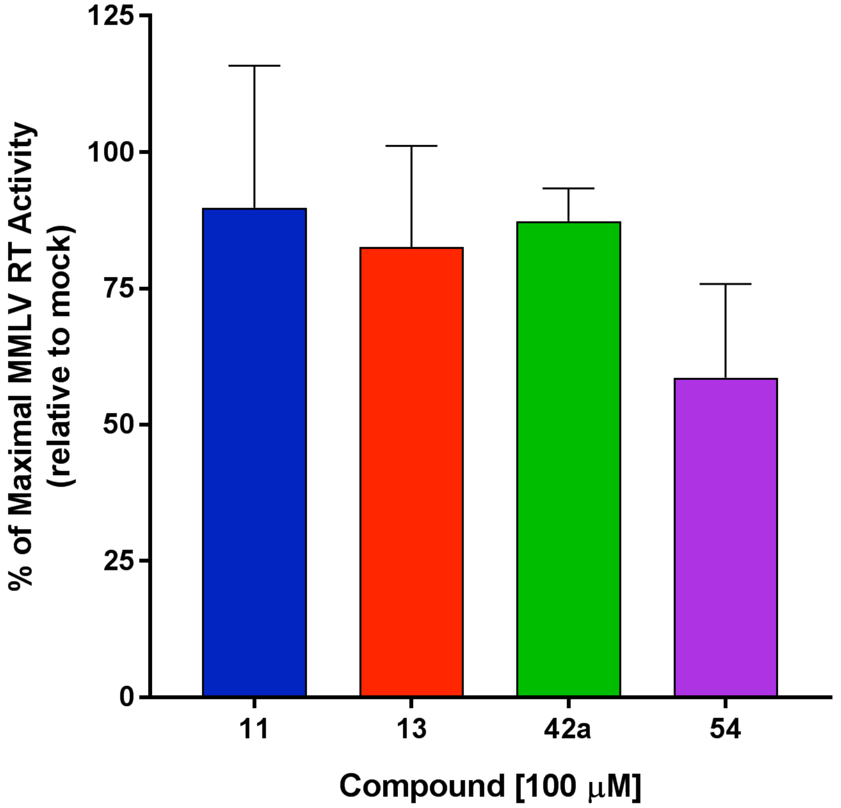

Supplementary Figure S4. Broad-spectrum activity screening of lead compounds. To test for specificity of calicivirus RdRp inhibition, the four lead compounds (**11, 13, 42a** and **54**) were examined at 100 μM for inhibitory activity against two additional polymerases. Panel A) bacteriophage Φ6 RdRp and Panel B) Moloney murine leukaemia virus reverse transcriptase (MMLV RT).

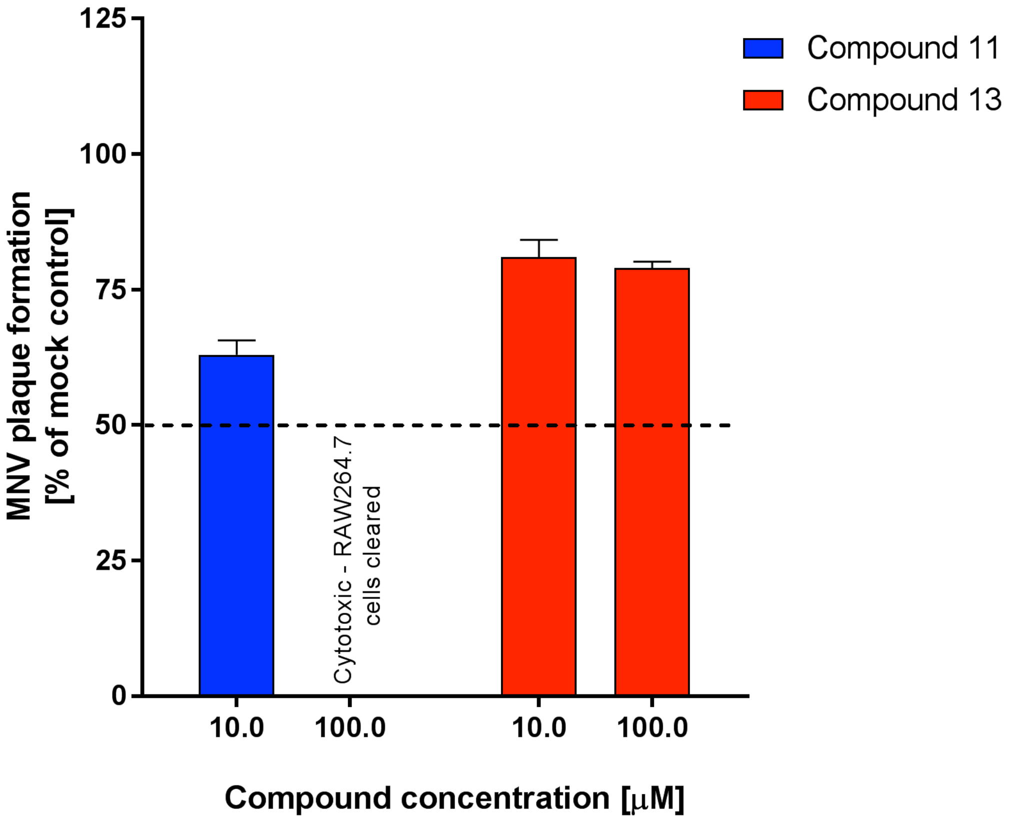

Supplementary Figure S5. Examination of compounds 11 and 13 in cell culture. Effects of compounds 11 and 13 (10 and 100 μM) were examined against MNV in a plaque reduction assay. The mean values of triplicate datasets are shown with standard deviations.

References

1. Mitsch, A. et al. Non-thiol farnesyltransferase inhibitors: *N*-(4-tolylacetylamino-3-benzoylphenyl)-3-arylfurylacrylic acid amides. *Bioorg. Med. Chem.* **12,** 4585-4600 (2004).
2. Gaubert, G. *et al*. Discovery of selective nonpeptidergic neuropeptide FF2 receptor agonist. *J. Med. Chem.* **52,** 6511-6514 (2009).
3. Pomel, V. et al. Furan-2-ylmethylene thiazolidinediones as novel, potent, and selective inhibitors of phosphoinositide 3-kinase *γ*. *J. Med. Chem.* **49,** 3857-3871 (2006).
4. Conrad, W.E., Fukazawa R., Haddadin, M.J. & Kurth, M.J. The Davis–Beirut reaction: N^1,N^2-disubstituted-1*H*-indazolones via 1,6-electrophilic addition to 3-alkoxy-2*H*-indazoles. *Org.Lett*. **13,** 3138-3141 (2011).
5. Leung, P.S.-W., Teng, Y. & Toy P.H. Chromatography-free Wittig reactions using a bifunctional polymeric reagent. *Org.Lett*. **12,** 4996-4999 (2010).
6. Pospíšil, J. & Potáček, M. Microwave-assisted solvent-free intramolecular 1,3-dipolar cycloaddition reactions leading to hexahydrochromeno[4,3-*b*]pyrroles: scope and limitations. *Tetrahedron*. **63,** 337-346 (2007).
7. Xie, H. et al. Structure−activity relationships in the binding of chemically derivatized CD4 to gp120 from human immunodeficiency virus. *J. Med. Chem.* **50,** 4898-4908 (2007).
8. Kumar, B.R.P. *et al.* Synthesis, glucose uptake activity and structure–activity relationships of some novel glitazones incorporated with glycine, aromatic and alicyclic amine moieties via two carbon acyl linker. Eur. *J. Med. Chem.* **46,** 835-844 (2011).

9. Zhang, W., Xie, J., Rao, B. & Luo, M. Iron-catalyzed *N*-arylsulfonamide formation through directly using nitroarenes as nitrogen sources. J. Org. Chem.. **80,** 3504-3511 (2015).

10. Isaad, J. & Perwuelz A. Simple route to a novel class of pyrazolidine-3,5-dione based azo dyes. *Tetrahedron Lett*. **51,** 5328-5332 (2010).

11. Korolev, D.N. & Bumagin, N.A. An improved protocol for ligandless Suzuki–Miyaura coupling in water. *Tetrahedron Lett*. **47,** 4225-4229 (2006).
